# The quest for nanoparticle-powered vaccines in cancer immunotherapy

**DOI:** 10.1186/s12951-024-02311-z

**Published:** 2024-02-14

**Authors:** Zhe Sun, Hui Zhao, Li Ma, Yanli Shi, Mei Ji, Xiaodong Sun, Dan Ma, Wei Zhou, Tao Huang, Dongsheng Zhang

**Affiliations:** 1 Department of Stomatology, Shandong Provincial Hospital Affiliated to Shandong First Medical University, Jinan, 250021 Shandong China; 2 Department of Endodontics, East Branch of Jinan Stomatological Hospital, Jinan, 250000 Shandong China; 3 Department of Endodontics, Gaoxin Branch of Jinan Stomatological Hospital, Jinan, 250000 Shandong China; 4 Department of Biomedical Engineering, Graeme Clark Institute, The University of Melbourne, Parkville, VIC 3010 Australia

**Keywords:** Nanoparticles, Immunotherapy, Cancer, Adjuvants, Innate immunity, Humoral immunity, Antigen

## Abstract

Despite recent advancements in cancer treatment, this disease still poses a serious threat to public health. Vaccines play an important role in preventing illness by preparing the body's adaptive and innate immune responses to combat diseases. As our understanding of malignancies and their connection to the immune system improves, there has been a growing interest in priming the immune system to fight malignancies more effectively and comprehensively. One promising approach involves utilizing nanoparticle systems for antigen delivery, which has been shown to potentiate immune responses as vaccines and/or adjuvants. In this review, we comprehensively summarized the immunological mechanisms of cancer vaccines while focusing specifically on the recent applications of various types of nanoparticles in the field of cancer immunotherapy. By exploring these recent breakthroughs, we hope to identify significant challenges and obstacles in making nanoparticle-based vaccines and adjuvants feasible for clinical application. This review serves to assess recent breakthroughs in nanoparticle-based cancer vaccinations and shed light on their prospects and potential barriers. By doing so, we aim to inspire future immunotherapies for cancer that harness the potential of nanotechnology to deliver more effective and targeted treatments.

**Graphical abstract:**

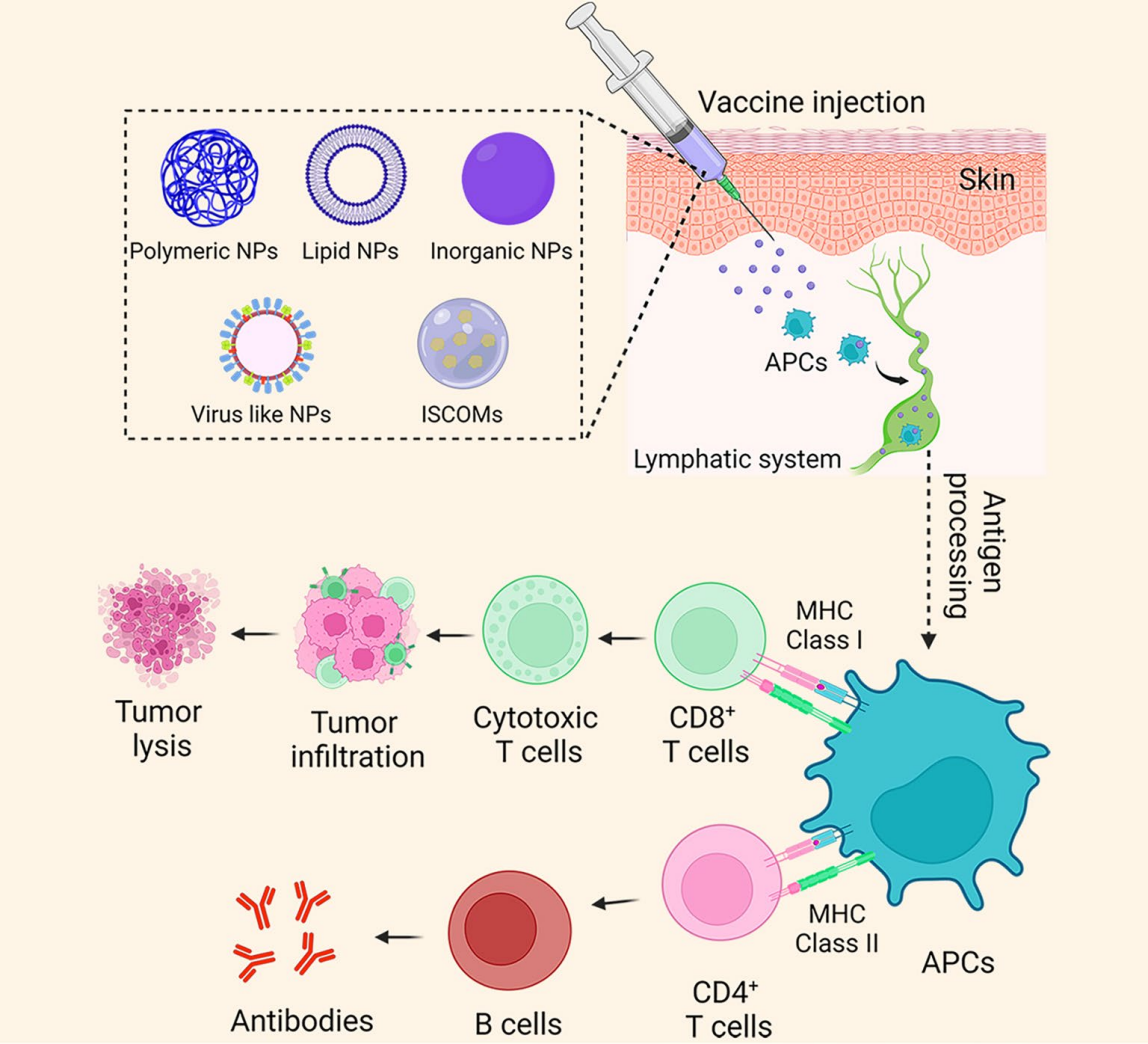

## Introduction

Vaccines stand as one of the most crucial tools for protecting people from infectious diseases and cancers [1–6]. Since Edward Jenner discovered the first vaccine (derived from the *Orthopoxvirus* cowpox species) to trigger protective immune responses against smallpox (*Vaccinia virus*) in 1796, significant progress has been made on vaccines development to save hundreds of millions of lives [7]. Beyond their success in preventing infectious diseases, vaccines have demonstrated immense potential in cancer immunotherapy by stimulating the immune system [8–11]. Both therapeutic and preventive cancer vaccines play a pivotal role in activating immunity against tumors caused by cancer cell mutations [12–14]. As we continue to unravel the complexities of the immune system and refine vaccine technologies, the prospects for leveraging vaccines in the battle against cancer become increasingly promising [15–17].

Although vaccines for treatment show great promise, most clinical research in this area is still in its early stages [18, 19]. One of the primary limitations of current cancer vaccines is their inability to elicit a sufficiently robust immune response against cancer cells [20–22]. To address this challenge, ongoing research explores various strategies, including virus-modified tumor vaccines, dendritic cell-based vaccines, DNA vaccines, protein vaccines, and peptide-based vaccines, as well as combinations of these strategies [23–31]. Among these approaches, peptide-based vaccines have emerged as the most commonly used ones [32–36]. Traditional vaccines, such as live-attenuated, inactivated, subunit, and conjugate vaccines [37–39], are not ideally suited for cancer vaccination due to their lack of specificity in distinguishing between normal host cells and cancerous host cells [40]. In contrast, peptide-based vaccines deliver peptide epitopes from shared tumor-associated antigens (TAAs), specifically targeting histocompatibility complex class I restricted peptides to activate CD8^+^ T cells against cancer [41, 42]. This specificity has positioned peptide-based vaccines as highly promising compared to classical vaccines [43]. However, these methods are limited by the fact that the antigen itself is unable to cross the cell membrane, and peptide antigens are prone to degradation by endogenous proteases [44]. To overcome these challenges, nanotechnology offers a potential solution by providing techniques to effectively deliver antigens to the desired sites [45, 46]. By harnessing nanotechnology, researchers aim to enhance the efficacy and precision of cancer vaccines, ultimately advancing the field of cancer immunotherapy [47–50].

The use of nanomaterials has provided new opportunities for enhancing the therapeutic effectiveness of cancer vaccinations [51–59]. Nanovaccines, in comparison to conventional vaccine formulations, offer distinct advantages, including prolonged release time, targeted delivery, and increased immunogenicity and antigenic stability [60, 61]. Notably, nanoparticles (NPs) stand out due to their tunability, allowing them to be customized in shape and size to suit various applications [62, 63]. Their exceptional physicochemical properties, such as large surface area-to-volume ratios, controllable surface charges, make them highly versatile delivery vehicles for vaccine formulations [60]. Moreover, NPs can be engineered with various targeting molecules including peptides, proteins, polymers, cell-penetrating peptides, and others on their surface [60, 64, 65], enabling efficient targeting and penetration of major components in the tumor microenvironment (TME) [66]. Furthermore, due to damaged lymphatic drainage and leaky tumor vasculature, NPs tend to accumulate more in tumors than in normal tissues, which significantly enhances the efficacy of nanovaccines [66]. NPs have been studied by several research groups with great success in the field of vaccination [51, 67–69]. Despite well-documented research on nanovaccine synthesis and applications, the majority of which discusses their possible use in treating diseases, infections, and other health issues [38, 70–73], there are few reports on nanovaccines in cancer immunotherapy [74, 75]. For example, Bhardwaj et al*.* summarized the use of nanovaccines in the treatment of infectious and non-infectious diseases, such as malaria, tuberculosis, human immunodeficiency virus (HIV)/AIDS, influenza, and cancer [70]. Zhou and his group members examined the current status of cervical cancer immunotherapy using therapeutic vaccines and adoptive cell therapies [74]. In this review, we firstly summarize the immunological mechanism of cancer vaccines; and then focus on the recent advancements of various types of NPs (polymeric NPs, lipid carriers, inorganic NPs, virus-like particles and immunostimulating complexes) in the field of cancer immunotherapy. Additionally, the benefits and disadvantages of these vaccines are discussed. Further, we will discuss the challenges and prospects of combining nanotechnology with other types of therapy. A literature review was conducted using Scopus, PubMed, and Web of Science to find articles mainly from 2018 to 2023, but also some important studies from 2010 on nanovaccines for cancer immunotherapy. By discussing the challenges and opportunities associated with nanovaccines, we aim to inspire future immunotherapies for cancer that harness the potential of nanotechnology to deliver more effective and targeted treatments.

### The immune system and nanoparticle vaccine

A person's immune system consists of both innate and adaptive responses [66]. NP vaccine is designed to primarily stimulate the adaptive immune response, which leads to effective and long-term immunogenicity [76]. To achieve this, vaccines need to be initially recognized by the host defense system, which then triggers an immune response [77].

Innate immune responses serve as the first line of defense against pathogens, providing a rapid and non-specific response upon infection [78]. After damage caused by a pathogen, the innate immune system activates within a few minutes to counteract the invasion [79]. These responses orchestrated by cellular effectors, including macrophages, neutrophils, dendritic cells (DCs), and natural killer (NK) cells, as well as other soluble factors like complement cascade proteins [78]. Various cells, including antigen-presenting cells (APCs) and mucosal/oral epithelial cells, express pattern recognition receptors (PRRs), such as Toll-like receptors (TLRs) [80], which are involved in inducing and enhancing both innate and adaptive immune responses [80, 81]. The adaptive immune system plays a crucial role in providing long-lasting protection against pathogen, although it may take several days to mount a full response [82]. Adaptive immunity encompasses both humoral immunity and cell-mediated immunity, both of which are essential for eliminating pathogens completely [82, 83]. Humoral immunity involves the production of antibodies by B lymphocytes in response to foreign antigens [84]. Cell-mediated immunity primarily involves CD4^+^ and CD8^+^ T cells, which are activated by APCs. CD4^+^ T cells can be classified into two types: Th1 cells, which support cellular immunity, and Th2 cells, which support humoral immunity [85]. The CD8^+^ T cells play a critical role in directly eliminating cancer cells and combating intracellular infections [84]. Their ability to recognize and destroy cancerous cells is instrumental in immune surveillance and defense against these threats.

APCs are a critical component of the innate immune system responsible for capturing, processing, and presenting antigens to B and T cells, leading to the stimulation and activation of humoral and cellular immune responses, respectively [86]. APCs mature during their migration to secondary lymphoid organs, becoming capable of activating naive T cells (CD4^+^ and CD8^+^) by presenting antigens on their surface as peptide/ major histocompatibility complex (MHC)-class I/II complexes [87]. The interaction between antigens and the T cell receptor (TCR), along with co-stimulatory signals, is essential for the stimulation of naive T cells [88, 89]. Co-stimulatory signals are released by CD28 on T cells bound to the CD80/86 on DCs. This interaction results in the proliferation and differentiation of naive T cells into effector cells [88, 89]. Extracellular antigens are typically presented by MHC-class II molecules on DCs, leading to the activation of CD4^+^ T cells [88]. On the other hand, cytosolic antigens are presented by MHC-class I molecules on DCs, leading to the activation of CD8^+^ T cells, also known as cytotoxic T cells [90]. Additionally, antigens released after lysis of infected cells can be captured by bystander DCs and presented to MHC-class I molecules to CD8^+^ T cells, which is termed antigen cross-presentation and plays a vital in generating an effective cancer vaccine [90, 91].

Cytotoxic T lymphocytes (CTLs) are crucial for eradicating cancers [92]. CTLs play a pivotal role in the adaptive immune system, possessing the ability to selectively eliminate target cells using various mechanisms, such as the release of cytokines, granzymes, and perforin. Additionally, CTLs can also induce target cell apoptosis through interactions with Fas and Fas ligand (FasL). Thus, vaccination can elicit broad endogenous antigen-specific CTLs to treat cancer [93]. A CTL response can be divided into four phases, which includes effector, contraction, immunological memory and a quick recall response [94]. DCs present antigen in the context of MHC class I to CD8^+^ T cells, which is crucial for the activation of naïve and memory CD8^+^ T cells [95]. There are three signals for the CD8^+^ T cell to develop an optimal CTL response during this process [94]. The first signal is elicited by the TCR/peptide-MHC class I interactions. The second signal comes from several co-stimulatory receptors/ligands, which are expressed by the activated DC and CD8^+^ T cell. The third signal is delivered via IL-12 or type I interferons (IFN) or through inflammatory signals from TLR ligands, finally leading to the required CTL response [94, 96]. The two major mechanisms that are involved in CTL response are via granule exocytosis (perforin and granzymes), or by the induction by death ligands/death receptor system [97]. Once the CTL response is stimulated, granules are quickly secreted by the microtubule-organizing center to the presynaptic membrane [97]. Granules then fuse with the plasma membrane and release perforin and granzymes, resulting in target cell death [97]. For the death ligands/death receptor system, after CTL activation, the expression of death ligands on the CTLs cell surface such as Fas ligand or TNF-related apoptosis-inducing ligand (TRAIL) would increase, which can destroy susceptible cancer cells by interaction with death receptors [97, 98]. It is generally believed that for the induction of effective long-lived CD8^+^ T cells, CD4^+^ T cell help is essential for APCs activation and the resulting production of IL-2 and IFN-γ [99, 100]. Furthermore, CD4^+^ T cells also help CD8^+^ T cells maintain and infiltrate at a tumor site by rendering the tumor environment permissive [100]. As a result, activation of both CD4^+^ and CD8^+^ T cell responses are essential to induce an effective antitumor immune response [101].

**Fig. 1:**
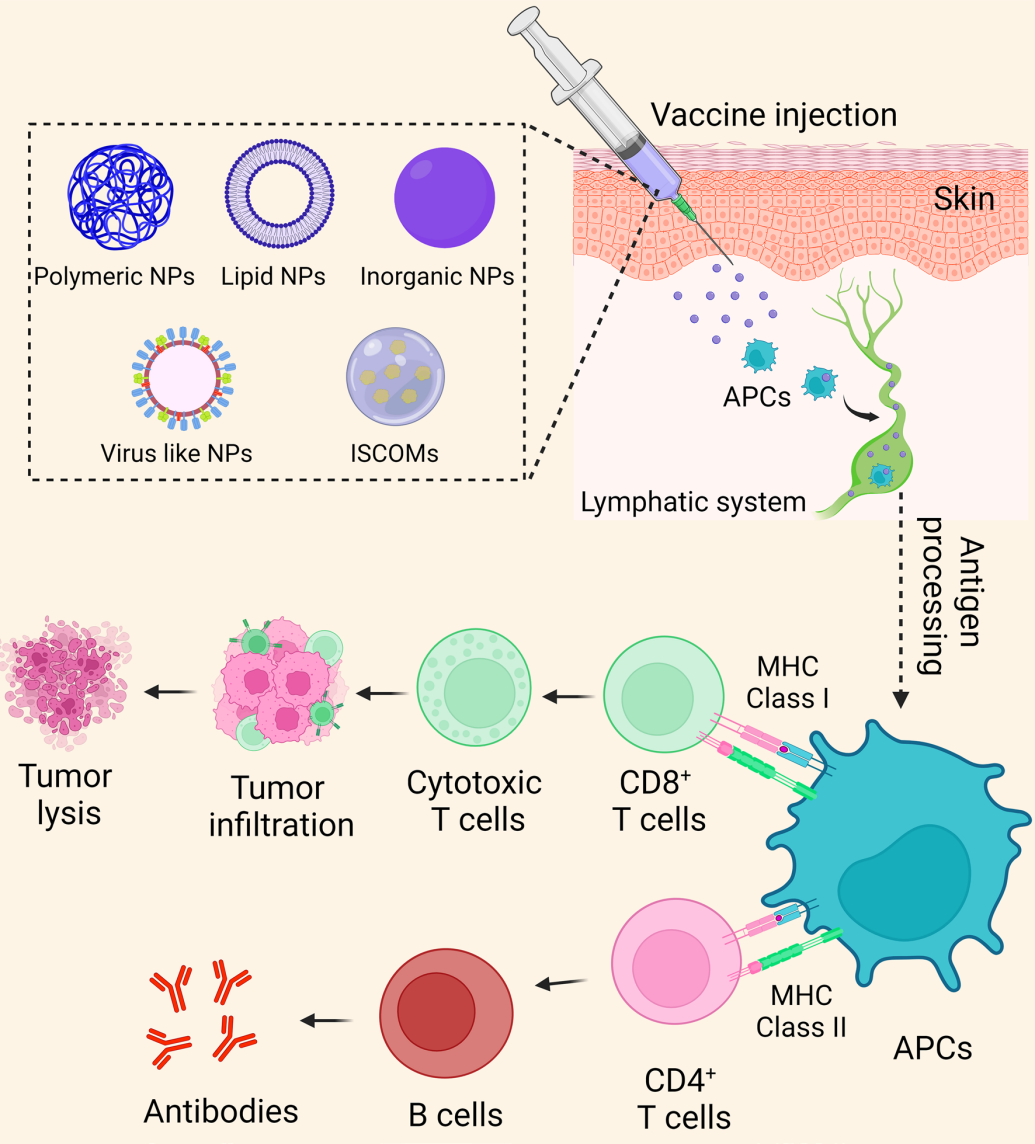
Nanoparticle vaccine for activation of the immune system. A variety type of NPs such as lipid-based vehicles, polymer-based vehicles, inorganics-based vehicles, and bio-inspired vehicles are used in vaccine formulation. NP cores with antigen are protected against enzymatic degradation, while surface immobilization mimics pathogen presentation of antigen. Antigens delivered with NPs are recognized by APCs and processed inside, inducing T cell responses. When the immune system is stimulated, CD8^+^ T cells are capable of recognizing tumor antigens and killing malignant cells. Moreover, provoking B cells leads to the secretion of antibodies and the activation of humoral immunity. The illustration was made using Biorender. NPs, nanoparticles; ISCOMs, immunostimulatin complexes; APCs, antigen-presenting cells; MHC, major histocompatibility complex

Tumor antigens can be loaded with NPs to activate an immune response (Fig. 1) [102]. NP cores with antigen are protected against enzymatic degradation, while surface immobilization mimics pathogen presentation of antigen [102]. Antigens delivered with NPs are recognized by APCs and processed inside, inducing T cell responses. When the immune system is stimulated, CD8^+^ T cells are capable of recognizing tumor antigens and killing malignant cells. Moreover, provoking B cells leads to the secretion of antibodies and the activation of humoral immunity [102].

Cancer immunotherapy works by stimulating the immune system and inhibiting immunosuppressive pathways, activating cytotoxic T cells, inhibiting tumor growth, and eliminating cancer cells [103]. The efficacy of cancer vaccinations is hampered by the tumor microenvironment and other immunosuppressive factors. The combination of cancer vaccines and nanotechnology will be an excellent strategy to induce potent antitumor responses [104]. With NPs as cancer vaccines, there are several advantages over traditional vaccines, including (1) protecting vaccines from degradation; (2) increasing the stability of antigens through the package shielding effect of carrier materials; (3) utilizing ligands to target DCs; (4) enhancing immunogenicity with immunological adjuvants such as exosomes and plant-derived immunoadjuvants; (5) strengthening the retention of antigens and adjuvants within lymph nodes by modifying their size and target specificity; (6) promoting cross-presentation to induce CTLs; and (7) controlling release and distribution [104, 105]. By facilitating antigen presentation and immunogenicity, nanotechnology can be used to greatly improve the delivery efficiency of cancer vaccines and to induce immune responses.

### Nanoparticles in cancer immunotherapy

Over the past decade, cancer immunotherapy has emerged as an effective strategy for harnessing the patient's immune system to fight cancer [11, 105–107]. In recent years, the use of nanomaterials has shown considerable promise in enhancing the effectiveness of cancer immunotherapy while mitigating undesired adverse effects [76, 106, 108–112]. A wide variety of NP delivery systems have been utilized as vaccine carriers and adjuvants, offering advantages over existing approaches [113–116].

Using NPs for cancer vaccines has many advantages that make it a promising approach in the field of cancer immunotherapy. NPs can protect fragile cancer antigens from degradation in the blood, increasing their stability and ensuring that they reach their intended target intact [117]. NPs can also effectively deliver cancer-specific antigens to immune cells, such as dendritic cells, which are essential for initiating an immune response against cancer cells [118]. This targeted delivery ensures that the immune system recognizes the cancer cells as foreign invaders. Besides, NPs can be designed to specifically target the tumor site, which reduces the risk of off-target effects, minimizes damage to healthy tissue, and improves the safety and effectiveness of cancer vaccines [119].

The integration of nanotechnology into chimeric antigen receptor T-cell (CAR-T) immunotherapy serves as an exemplary illustration. While CAR-T therapy has demonstrated success in addressing hematologic tumors [120], its application to solid tumors faces challenges such as limited efficacy, off-target effects, and elevated costs [121]. These challenges stem from the constrained infiltration ability of CAR-T cells into solid tumor cells, coupled with complications like cytokine release syndrome and CAR-T-associated encephalopathy syndrome [122]. Notably, nanotechnology has proven transformative in CAR-T immunotherapy, playing a pivotal role in CAR-T cell construction, transfection, expansion, delivery, and subsequent anti-tumor effects [123, 124]. Leveraging nanoscale materials has significantly improved the precision and efficacy of CAR-T immunotherapy, offering solutions to longstanding challenges in cancer treatment [125]. Key advancements include the utilization of nanocarriers, such as lipid NPs and polymer systems, facilitating the targeted delivery of CAR-T cells to tumor sites, thereby minimizing off-target effects, and enhancing therapeutic outcomes [126]. Furthermore, the incorporation of nanomaterials augments the engineering functionalities of CAR-T cells, enhancing persistence and regulating the release of therapeutic payloads. This synergistic interplay between nanotechnology and CAR-T immunotherapy not only amplifies the therapeutic potential of this approach but also paves the way for the development of the next generation of nanoscale cancer vaccines. This review will not delve into the nanotechnology used in CAR-T therapy as it has been comprehensively reviewed elsewhere [127–129].

The merits of NPs make them an exciting avenue for developing innovative cancer immunotherapies. The release kinetics of antigens can be controlled through the design of NPs [77]. A sustained release can stimulate a more durable and robust immune response, which may be necessary to eradicate cancer cells [130]. The immune response generated by nanoparticle vaccines can lead to the formation of durable immune memories that may provide protection against cancer recurrence. Additionally, NPs can be designed to carry a variety of ingredients, such as antigens, adjuvants, and even therapeutic drugs [131]. This versatility allows for a comprehensive approach to cancer treatment that targets the response of cancer cells and the immune system. Some NPs themselves can act as adjuvants, which can enhance the immune system's response to cancer antigens [132]. By delivering antigens and adjuvants directly to immune cells, NPs can reduce systemic exposure to these components, potentially reducing the risk of toxic side effects. Moreover, NPs can readily be administered through conventional injection methods, simplifying their integration into clinical practice [133]. Additionally, NPs can be tailored to carry patient-specific cancer antigens, rendering them a platform for personalized cancer vaccines tailored to the unique characteristics of each patient's tumor [134]. These merits make NPs an exciting avenue for developing innovative cancer immunotherapies.

While NPs have several advantages as vaccine carriers/adjuvants, they currently face several disadvantages, which have limited their widespread use [135, 136]. A major challenge in NP use is reproducibly synthesizing homogeneous NPs of non-aggregated sizes and shapes [137, 138]. It has been shown that NPs aggregate rapidly in aqueous solutions, resulting in uncontrolled biological responses [139]. It is essential to synthesize NPs that are uniform in size, stable in aqueous solutions, and reproducible in production before using them in clinical settings [138]. For today's commercial applications, NPs with defined sizes and shapes need to be produced using a standardized method. To scale up commercially, the method must also result in NPs with low polydispersity, no post-synthesis aggregation, high yield, and high stability [137]. In addition, although nanomedicine is a rapidly developing field, there is currently little guidance available. It is a global problem that nanomedicines and nanomaterials are not regulated on a formal basis for health-related purposes [140]. A major problem in the regulatory process for nanomedicines is that regulatory agencies such as US Food and Drug Administration (FDA) rely on bulk material safety data, which does not display similar pharmacodynamic and pharmacokinetic properties [141]. As a result, once nanomedicine has received marketing authorization, its safety and efficacy data may not accurately reflect clinical experience. It is also challenging to classify nanomedicines [141]. Furthermore, NP vaccines have the potential to induce adverse local inflammatory responses [142]. Most importantly, the biodegradability and solubility of nanomaterials are always a concern [77]. As a result, more research is needed to develop effective and safe NP-based vaccines.

A variety of nanomaterial-based delivery vehicles, such as polymers, lipids, inorganics, and bio-inspired vehicles, bring unique benefits to the development of cancer vaccines [12, 52, 102, 143, 144].

### Polymeric nanoparticles

Polymeric NPs are highly appealing as vaccine carriers due to their adjuvant properties [145–149]. They possess desirable characteristics such as biodegradability, water-solubility, non-toxicity, and cost-effectiveness [150, 151]. Additionally, cationic polymers are able to enhance stability, enabling them to withstand cellular trafficking [150]. Both synthetic and natural polymers can be used to form polymeric NPs, such as chitosan (natural), poly (lactic-co-glycolic acid) (PLGA) and poly (lactic acid) (PLA) (synthetic) [152]. By manipulating NP properties such as shape, size, charge, hydrophobicity, polymer composition, and concentration, the loading capacity of antigens and the rate of polymer biodegradation can be controlled [153].

Among various polymeric NPs, PLGA NPs are the most extensively investigated as vaccine carriers, largely due to their US FDA approval and licensing for medical applications [73, 153–155]. An early study has shown that PLGA NPs loaded with indocyanine green (ICG) and a toll-like-receptor-7 agonist (R837), combined with photothermal therapy (PTT), can elicit a greater anti-tumor immune response compared to traditional adjuvants [156]. The integration of PTT and immunotherapy exemplifies a synergistic paradigm within cancer treatment [157]. PTT employs light-absorbing NPs to convert absorbed light into heat, thereby damaging cancer cells. This localized thermal impact not only directly targets cancer cells but also triggers a cascade of immunogenic changes, fostering the release of tumor-associated antigens and facilitating the recruitment of immune cells [158]. When coupled with immunotherapies designed to invigorate the body's immune system to recognize and eliminate cancer cells, this dual strategy forms a potent alliance. PTT-generated heat enhances tumor immunogenicity, refining the immune system's capacity to recognize cancer cells and promoting the activation and infiltration of immune cells. Concurrently, immunotherapy augments the anti-cancer immune response systemically, potentially addressing metastatic or residual tumor cells [159]. This integrative approach holds the promise of enabling a more thorough and effective cancer treatment, capitalizing on the strengths of both methodologies to achieve superior treatment outcomes. In a comparative research study on cationic liposomes and PLGA NPs, it was demonstrated that synthetic long peptide-loaded cationic liposomes and PLGA NPs induced greater production of T cells in vivo when compared to Montanide ISA 51- and squalene-based emulsions, making them strong candidates for cancer immunotherapy [160]. In another study, Zuo and co-workers developed a tumor vaccine using Dermatophagoides protein 1 (Der p1) encapsulated in PLGA NPs, which notably inhibited the growth of Lewis lung cancer cells in a mouse model by activating the generation of Th1 cytokines (IFN-γ and IL-4) [161]. Similarly, in a mouse model of diphtheria and tetanus, when diphtheria and tetanus toxoids (DTaP) antigens were adsorbed into PLGA NPs and co-delivered with a TLR7 ligand, enhanced production of immunoglobulin G (IgG) and IgG2a antibodies was observed, highlighting the potential of PLGA NPs as a potent adjuvants for vaccine formulation [162]. Several modifications have been explored to improve the effectiveness of PLGA NPs as vaccine carriers. For instance, mannose-functionalized PLGA NPs designed to target melanoma cancer demonstrated that PLGA NPs with a diameter of 150 nm encapsulating MHC class I- or class II-restricted melanoma antigens and TLR ligands (Poly (I:C) and cytosine-phosphate-guanine (CpG)) exhibited the highest tumor growth delay [163]. Hu et al. demonstrated that conjugating higher concentrations of cholesterol to PLGA NPs resulted in better-controlled antigen release, increased uptake by dendritic cells, and improved antigen stability compared to lower cholesterol concentrations [164]. Xu et al*.* designed pH-sensitive PLGA NPs loaded with astragalus polysaccharide (APS) as an adjuvant system to enhance immune responses [165]. Their results revealed that pH-responsive APSPs considerably increased macrophage phagocytosis capacity and markedly increased MHC-II, CD80, and CD86 expression [165]. When compared to APS alone, both OVA-loaded NPs were able to dramatically increase the proliferation, differentiation, and maturity of mouse spleen lymphocytes and dendritic cells, respectively, as well as trigger stronger Th1-biased immune responses. NPs dramatically increased the production of TNF-α, IL-4, IL-6, IFN-γ, and antigen-specific IgG antibody responses [165].

Despite these advantages, PLGA NPs have some limitations. Their short half-life often leads to rapid degradation, resulting in the loss of immunogenicity and effectiveness. Consequently, the vaccine may require more booster injections to sustain immune responses, thus compromising long-term protection. Future efforts should focus on addressing these disadvantages to optimize the potential of PLGA NPs [150, 166].

**Fig. 2:**
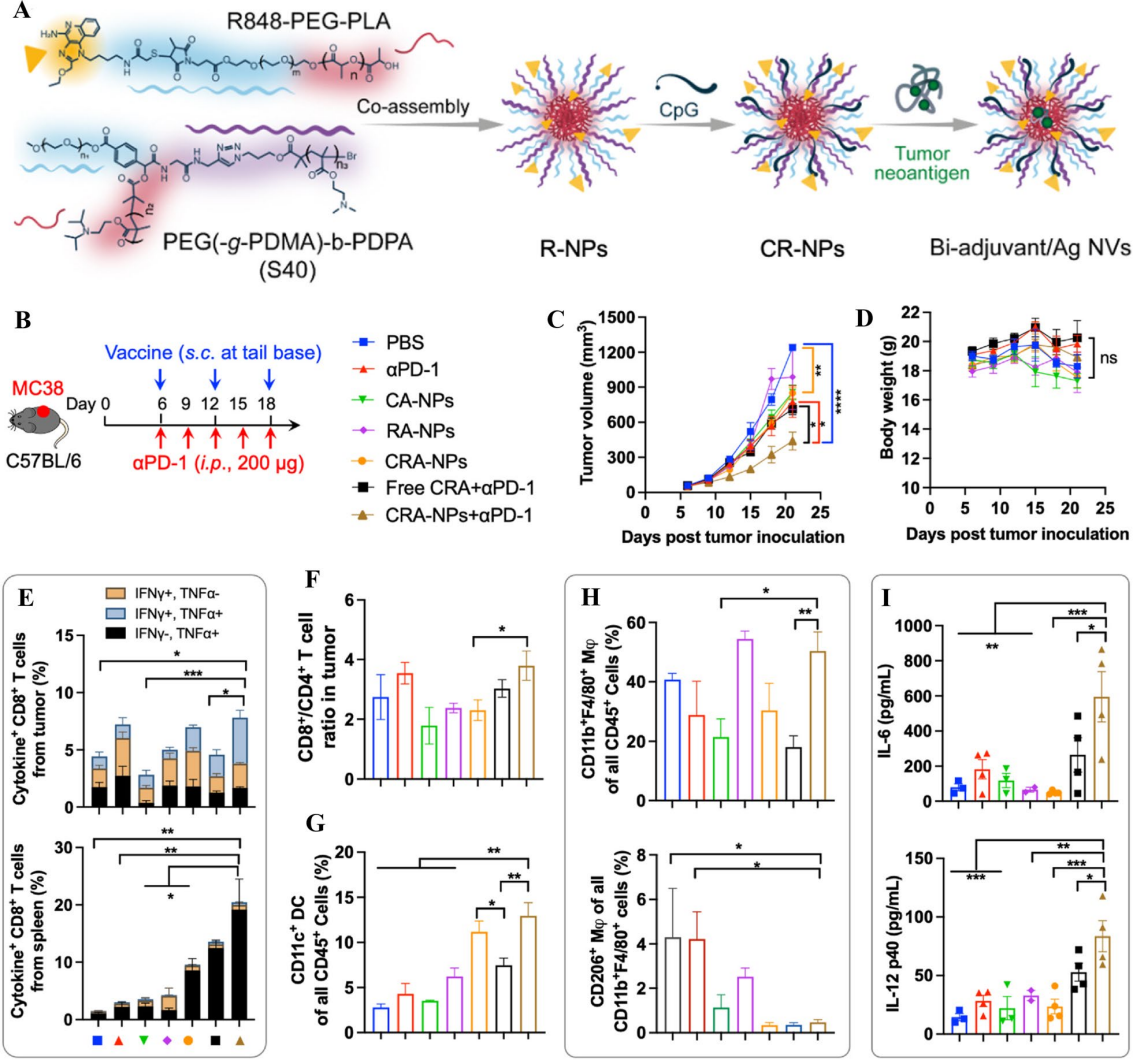
The use of polymeric nanoparticles for the delivery of bi-adjuvants and neoantigens for cancer immunotherapy. **A** A pH-responsive ionizable polymer, PEG(-g-PDMA)-b-PDPA or S40, was loaded with two immunostimulant adjuvants, R848 and CpG, together with cancer neoantigen peptides. **B** Study design for MC38 cancer immunotherapy. **C** The growth curves of MC38 tumors. **D** The mouse body weights after treatment in MC38-bearing C57BL/6 mice. **E** The immunocytochemistry of CD8 ^+^ T cells in the spleen (lower) and tumor (upper) was measured by intracellular staining of IFN-γ and TNF-α on day 21. **F** Ratio of tumor infiltrating CD8 ^+^ /CD4 ^+^ T cells on day 21 in mice treated as described above. **G** The percentage of CD45 ^+^ CD11c ^+^ DCs in intratumorally tumors on day 21. **H** The percentage of CD45 ^+^ CD11b ^+^ F4/80 ^+^ macrophages (Mφ) and CD206 ^+^ Mφ in tumor on day 21. **I** Secretion of IL-6 and IL-12p40 by mouse splenocytes after incubation in 96-well plates for 12 h. Adapted with permission from ref [172]. Copyright (2023) Bioactive materials. PD-1, programmed death protein 1; CRA-NPs, CpG/R848/Adpgk-codeliverying nanoparticles; IL, interleukin

Another polymer that has been used in the development of NP vaccine delivery systems is PLA [167]. Similar to PLGA NPs, PLA NPs are biodegradable, non-toxic, and biocompatible, and PLA has also been approved by the US FDA for biomedical applications [154]. Research has indicated that PLA NPs have the potential to significantly enhance vaccine efficacy [168, 169]. Pavot et al. synthesized PLA NPs (200 nm) containing Gag p24 HIV-1 antigen, along with PRR domains (Nod)-like receptors 1 and 2. The results showed that PLA NPs were effectively taken up by DCs and led to increased production of pro-inflammatory cytokines (IL-6, IL-1β, TNFα, IFNγ and IFNα). Furthermore, compared to Alum (a commonly used vaccine adjuvant derived from aluminum salts) [170], the PLA NPs resulted in a 100-fold increase in the antibody response [168]. Other researchers utilized cationic polymer (including chitosan, chitosan chloride, and polyethylenimine) coated PLA microspheres with conjugated viral Hepatitis B antigen (HBsAg) to induce robust humoral and cell-mediated immune responses. They found that HBsAg adsorbed on PLA microspheres significantly increased antigen uptake and the expression of CD86, MHC I, and MHC II as well as the production of IL-1β, IL-6, TNF-α, and IL-12 in macrophages [169]. Interestingly, the same group also found that the route of vaccine administration influenced the efficacy of PLA NP vaccines, with intramuscular administration eliciting a stronger humoral and cell-mediated immune response compared to subcutaneous vaccination [171]. In a recent study, Su et al*.* developed and tested PLA NPs co-delivered bi-adjuvant (R848 and CpG) and neoantigen peptides (neoAgs) as well as immune checkpoint blockade (ICB) to induce antitumor immune response (Fig. 2) [172]. Compared to controls, the CD8^+^/CD4^+^ T cell ratio in TME was significantly increased by CpG/R848/Adpgk-codeliverying NPs (CRA-NPs) + an anti-programmed death-1 antibody (αPD-1), indicating a positive response to tumor therapy (Fig. 2F). Furthermore, intratumoral CD11c^+^DCs and CD11b^+^F4/80^+^ macrophages were increased by CRA-NPs + αPD-1, but the frequency of immunosuppressive M2-like CD206^+^ macrophages was decreased (Fig. 2G and H). These nanovaccines showed potent immunogenic characteristics by potentiating peptide antigen immunogenicity, eliciting robust antitumor immune responses with memory, and remodeling the tumor immune microenvironment with reduced immunosuppression (Fig. 2) [172]. Although PLA NPs have demonstrated the potential as efficient vaccine adjuvants, they are susceptible to deterioration under certain conditions, such as excessive heat, sonication, organic solvents, and freezing, which may lead to serious aggregation or degradation of antigens [154]. Additionally, the acidic monomers produced during polymer degradation can result in the degradation of the tertiary NP structure. PLA NPs can be strengthened through incorporating stabilized chemicals and surfactants or optimizing synthesis methods [154]. Further limitations of PLA NPs as vaccine candidates include low encapsulation efficiency and insufficient drug loading capacity, which need to be addressed for their potential use in the near future [173].

Chitosan is a natural cationic polymer derived from chitin [174]. It possesses several advantageous characteristics, including low cost, ease of manufacturing, biological origin, high biocompatibility and biodegradability [175]. These properties have facilitated the development of chitosan as a vaccine carrier in the past decade [176–179]. Chitosan has proven to be suitable for mucosal vaccine delivery and to be able to improve mucosal immune response [180]. Zhao and co-workers designed a chitosan-loaded NP vaccine incorporating the Newcastle disease viruses (NDV) through an ionic cross-linking strategy. Their findings demonstrated that chitosan NPs were safe and cost-effective compared to commercially attenuated NDV vaccines, and exhibited enhanced and expedited cellular immunity (increased IFN-γ production), humoral immunity (increased IgG production) and mucosal-immunity (increased IgA production) [181]. The increased production of IgA, a key immunoglobulin secreted by B lymphocytes, in response to chitosan exposure suggests its potential as an effective mucosal vaccine adjuvant [181]. Another recent study by Gheybi et al*.* showed that chitosan NPs encapsulating recombinant CD44 variants (rCD44v) induced a significant immune response in mice and provided protection against breast cancer in vivo [175]. The study utilized chitosan-rCD44v NPs (146.5 nm) and observed significantly higher levels of IgG and IgA in immunostimulant mice. Furthermore, compared to control groups, both injection and nano-injection test groups exhibited a notable reduction in tumor growth [175]. However, one of the major drawbacks of chitosan is the limited solubility in aqueous solutions, being only soluble in acidic solutions of low concentration inorganic acids and in pure organic solvents, which restricts its application in medical research [182, 183]. Despite its potential in improving vaccine efficacy, chitosan has not yet been used in an adjuvant in human studies, nor has it approval, nor has it been approved for human use, and currently there are no commercialized chitosan products on the market [184]. Even though chitosan can improve vaccine efficacy, there are still some potential problems that need to be addressed.

In summary, because of their excellent adjuvant qualities, polymeric NPs are very attractive as vaccine carriers. They have favorable qualities such being non-toxic, biodegradable, soluble in water, and economical [185]. Most polymers employed in formulation science are biodegradable and low toxicity, which makes them perfect for delivering a variety of medicinal chemicals. Polymer-based materials are attractive and unique, which makes them perfect antigen delivery platforms.

### Lipid nanoparticles

**Fig. 3:**
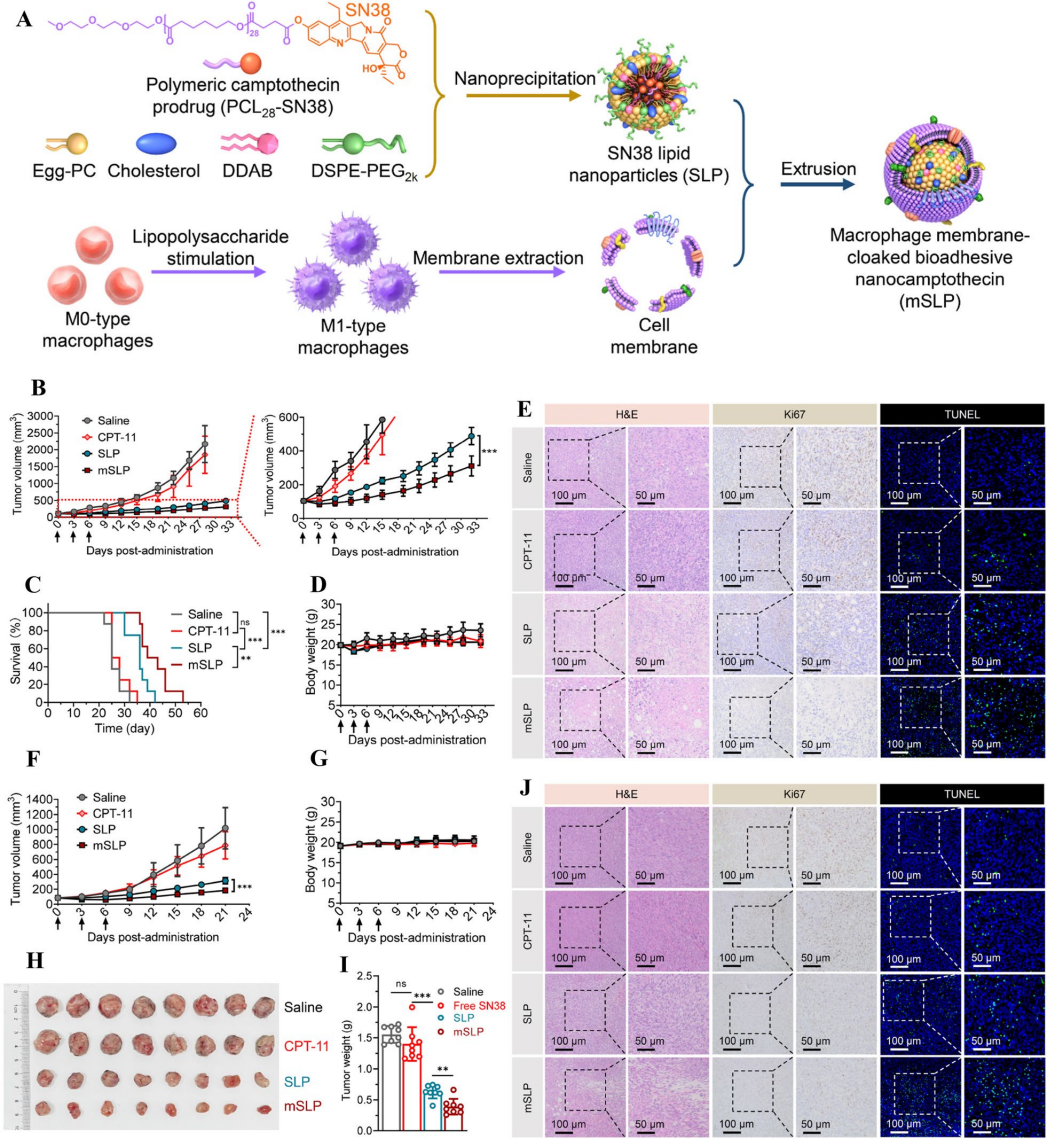
A biomimetic adhesive polycaprolactone nanocamptothecin based on macrophage membranes for improved cancer-targeting efficiency and metastasis inhibition. **A** The diagram illustrates the procedure for preparing macrophage membrane-camouflaged polymeric nanotherapy (mSLP). Triple-negative breast cancer (TNBC) mouse models bearing 4T1 **B**–**E** and Py8119 tumors **F**–**J** were treated with NPs in vivo for antitumor activity. **B** Following different drug treatments, tumor progression curves in the 4T1 orthotopic tumor-bearing mouse model were analyzed (n = 8). **C** Mouse survival curves (n = 8) from different treatment groups. **D** The body weight of mice in each group was monitored (n = 8). (**E**) On tumor sections, H&E, Ki67, and terminal deoxynucleotidyl transferase dUTP nick end labeling (TUNEL) staining was performed. **F** After different drug treatments, tumor progression curves are shown in the Py8119 orthotopic tumor-bearing mouse model (n = 8). **G** Each group's body weight (n = 8) was monitored. (**H** and **I**) A photograph and weight of excised tumors from each group at the study's end. **J** Tumor sections stained with H&E, Ki67, and TUNEL. Adapted with permission from ref [67]. Copyright (2023) Bioactive materials. DDAB, dimethyldioctadecylammonium bromide; DSPE-PEG_2k_, 1,2-Distearoyl-sn-glycero-3-phosphoethanolamine-N-[methoxy (polyethylene glycol) 2000]; SLP, SN38 lipid nanoparticles; FITC, fluorescein isothiocyanate; TNBC, Triple-negative breast cancer; TUNEL, terminal deoxynucleotidyl transferase dUTP nick end labeling

Lipid carriers have been extensively explored as drug vectors in the past few decades [186–189]. In recent years, there has been a notable increase in research exploring their potential use as vaccine carriers [51, 72, 190–195]. The most commonly utilized lipid NPs are liposomes, which are vesicular structures composed of lipid bilayers and an aqueous inner component [196]. Liposomes have many advantages over other vaccine delivery systems, including biocompatibility, the ability to encapsulate various agents, versatility, and plasticity [196]. Their synthesis flexibility allows for modification of the lipid composition to achieve a variety of properties such as size, charge, and the ability to encapsulate lipophilic component or hydrophilic antigen [153]. Swaminathan et al*.* demonstrated that lipid NPs, when combined with a TLR9 agonist, can act as potent subunit vaccine carriers, inducing significant CD4^+^ and CD8^+^ T cell responses to Ovalbumin (OVA) [197]. Another study showed that, modified lipid NPs incorporating a tumor metastasis targeting (TMT) peptide significantly inhibited tumor metastasis progression and lengthened the survival time of mice in a mouse cancer model, indicating the potential of lipid NP vaccines in preventing tumor metastasis [198]. Similarly, Ying et al. conducted a study in 2023, where nanocamptothecin derived from macrophage membranes boosted cancer-targeting efficiency and inhibited metastasis, and suppressed tumor growth without causing systemic side effects (Fig. 3). They developed a polymer-conjugated camptothecin prodrug that was encapsulated in macrophage plasma membranes stimulated with lipopolysaccharide. Through polymer conjugation, the parent camptothecin agent (e.g., 7-ethyl-10-hydroxy-camptothecin) was revived and lipid NPs were encapsulated. The results showed as compared to SN38 lipid NPs (SLP), M1-type macrophage membrane-cloaked cytotoxic nanocamptothecin therapy (mSLP) showed superior activity in inhibiting tumor progression. Furthermore, the mSLP treatment group displayed significantly improved survival rates (Fig. 3C). The membrane-cloaked nanocamptothecin was significantly more effective than SLP at inhibiting tumor growth (Fig. 3F). Preclinical studies showed that macrophage-camouflaged nanocamptothecin accumulated more in tumors than uncoated NPs [67]. To optimize the characteristics of NPs, researchers have also synthesized lipid-polymer NPs that consist of a polymeric core and a lipid shell. These lipid-polymer NPs exhibited enhanced cellular uptake by DCs and protected antigens from elimination during circulation [199]. In a recent study in 2023, lipid NP functionalized with herpes simplex virus type 1 glycoprotein D and the self-amplifying mRNA induced memory T cell responses that prevented the relapse of subcutaneous tumors and provided strong tumor protection in mouse model [72]. Interestingly, lipid microparticles with larger size (1150 ± 100 nm) were found to elicit similar effects in cancer prevention when compared to nanoparticle (90.15 ± 2.92 nm and 300 ± 40 nm) [200].

Despite their advantages, lipid carriers face challenges with stability when they come into contact with serum, both in vitro and in vivo. Upon contact, liposomes can quickly leak encapsulated molecules, such as antigens, before being captured by APCs, thus limiting their efficacy in vaccine delivery [196]. Several strategies have been utilized to address the stability issues associated with lipid carriers. Researchers have optimized the lipid composition of NPs to improve their stability. This optimization process involves the careful selection of lipids that form robust structures, minimizing susceptibility to disruption when exposed to serum components. For example, the deliberate choice of saturated lipids, as opposed to unsaturated ones, serves to reduce the presence of oxidizable lipid groups in the membrane [201]. Combining lipid NPs with biocompatible polymers, typically poly-(ethylene glycol) (PEG), to produce sterically stabilized lipid carriers can improve the surface properties of the lipid carriers by preventing access to their surface through steric hindrance and avoiding phagocyte removal from the blood flow [202]. This modification can create a protective layer that shields the lipid components from interactions with serum proteins, preventing premature leakage of encapsulated molecules. The increased circulation half-lives of sterically stabilized lipid carriers also increase their passive accumulation in cancer tissues by the enhanced permeation and retention effect, further increasing their effectiveness [203]. Improved encapsulation techniques, such as thin film hydration and microfluidic methods, have been developed to achieve high encapsulation efficiency and enhance the retention of molecules within lipid NPs [204, 205].

Despite the challenges, the benefits of lipid NPs, such as their efficient delivery of payloads and ability to elicit strong humoral and cell-mediated immune responses, make them a valuable tool in vaccine development [206]. The continued success of vaccines utilizing lipid NPs underscores the effectiveness of these strategies in overcoming stability issues. Notably, the recent approval of two lipid-mRNA-based vaccines from Pfizer-BioNTech and Moderna by the US FDA for COVID-19 prophylaxis in emergency situations has led to a significant increase in market value and substantial interest in the application of mRNA lipid nanoparticle vaccines, particularly in the field of cancer [10, 71, 207].

Lipid NPs have many advantages over other vaccine delivery systems, including biocompatibility, the ability to encapsulate various agents, versatility, and plasticity [196]. Despite being unstable in physiological conditions, lipid NPs possess considerable potential as anticancer therapeutics [208].

### Inorganic nanoparticles

In recent times, there has been significant research focused on the utilization of inorganic NPs for cancer immunotherapy [12]. In contrast to organic nanomaterials, inorganic NPs offer various advantages and possess unique properties that are beneficial for cancer therapy [209]. One key advantage is their ability to control the synthesis process [210–212]. Among the inorganic NPs commonly employed in vaccines, gold, silver, silica and calcium phosphate are the four most frequently utilized types [12, 209].

#### Gold nanoparticles

Gold NPs (AuNPs) are considered promising candidates for vaccine development due to their highly modifiable surface, biocompatibility, physiologically stability, ease of manipulation and manufacturing [213–217]. Furthermore, with the assistance of specially functionalized molecules, they possess the ability to penetrate blood vessels and barriers and target specific cells [218, 219]. Moreover, AuNPs can promote the function of T lymphocytes and enhance antitumor immunity by cross-presenting antigens [220]. These remarkable characteristics position AuNPs as good candidates for cancer vaccine treatments [215, 218, 221, 222].

**Fig. 4:**
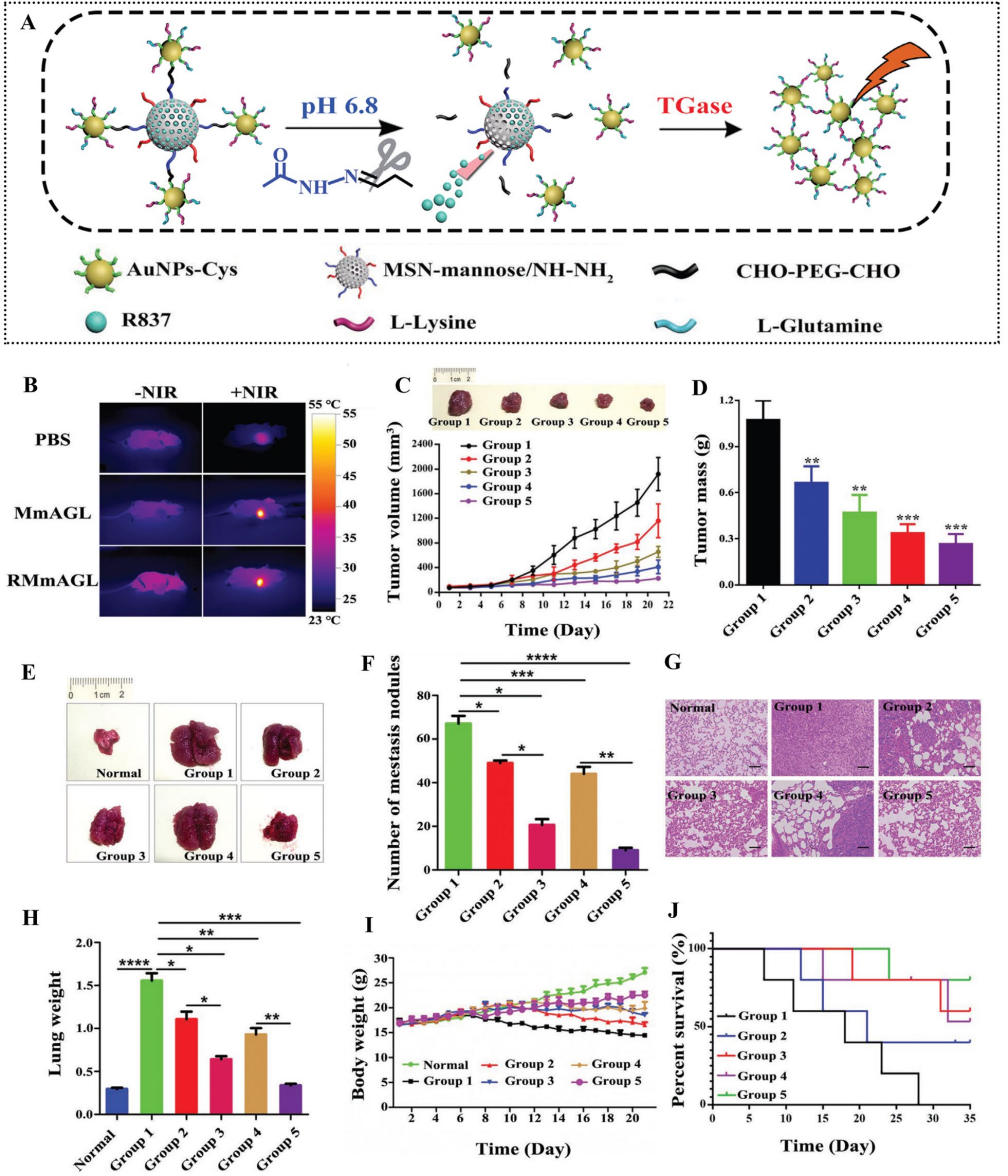
A novel multiresponsive adjuvant nanoparticle (R837@MSN-mannose- AuNPs-Glu/Lys) is fabricated to perform tumor-specific photothermal therapy while also working as a tumor-associated immune cell modulator for primary tumor eradication and prevent metastasis. **A** This schematic depicts the release of R837 and AuNPs-Glu/Lys for tumor-specific photothermal therapy in an acidic environment (pH 6.7), and the TGase-mediated aggregation of detached AuNPs-Glu/Lys. **B** Tumor-bearing mice treated with PBS, MmAGL, and RMmAGL combined with and without NIR irradiation are shown in vivo photothermal images. **C** A digital picture of the final tumor tissue and a graph showing the growth curve of the tumor after treatment with different formulations. **D** After 21 days of treatment with different formulations, tumor tissue mass was collected. **E** Following treatment with different formulations, BALB/c mice's lungs were photographed. **F** Different groups of lung samples were examined for metastatic nodules. **G** Histological images taken from different groups of lung samples undergoing H&E staining. **H** Different groups' average lung weights. **I** Curves of body weight in tumor-bearing mice treated with different formulations. **J** Tumor-bearing mice's survival percentages after treatments with different formulations. Adapted with permission from ref [221]. Copyright (2023) Advanced materials. AuNPs, gold nanoparticles; MSN, mesoporous silica nanoparticles; RMmAGL, Multiresponsive adjuvant nanoparticles; Glu, glutamine; Lys, lysine; Cys, cysteine; NIR, near-infrared. There are five groups: PBS, MmAGL, RMmAGL, MmAGL with NIR irradiation, and RMmAGL with NIR irradiation

Extensive testing and investigation have been conducted on the potential use of AuNPs in cancer nanomedicine [221, 223–228]. For example, AuNPs coated with OVA and the CpG adjuvant have been developed as a cancer vaccine. These coated AuNPs induced robust antigen-specific immune responses in a mouse tumor model, leading to significant antitumor activity and prolonged survival time. Notably, these antitumor responses occurred without the need of additional adjuvants, suggesting the competence of AuNPs as peptide vaccine delivery carriers [229]. A recent study by Dykman et al. found that the thermostable cancer antigen conjugated AuNPs (15 nm) prevented the development of xenografted tumors in mice. Mice immunized with complete Freund's adjuvant and AuNPs produced the highest titer, and after a 24-day period, no tumor growth was observed. Additionally, the production of proinflammatory cytokine (INF-γ, IL-6, and IL-1) was reduced compared to the mice immunized with other methods [230]. Similarly, other researchers have developed a cancer vaccine based on cytosine-phosphate-guanine (CpG) dinucleotides@AuNPs, which inhibited both primary and metastatic melanoma in mice by influencing CD8^+^ T cells and IFNγ production. Moreover, the vaccine treatment promoted the filtration of Th1 and CTL infiltration while stimulating the production of IFNγ and TNFα [231]. Currently, a novel approach in antitumor immunotherapy involves inhibiting autophagy with AuNPs [228]. In 2023, Zhang et al. successfully elicited potent antitumor immune responses by inhibiting the M2 polarization of tumor-associated macrophages (TAMs) through autophagy intervention with PEG-AuNPs. In both in vitro and in vivo models, PEG-AuNPs suppressed TAMs M2 polarization, triggered antitumor antibody production, and inhibited tumor growth in the subcutaneous region [228]. To effectively prevent tumor metastasis and recurrence, Liu et al. fabricated multiresponsive adjuvant NPs (RMmAGL) for tumor-specific photothermal therapy while controlling the activity of tumor-associated immune cells (Fig. 4). These NPs were made by combining mesoporous silica NPs (MSN) loaded with imiquimod (R837) and mannose (R837@MSN-mannose) with glutamine (Glu)/lysine (Lys)-commodified AuNPs through acid-cleavable hydrazone bonds [221]. The acidic tumor environment caused the separation of AuNPs-Glu/Lys from RMmAGL, resulting in the release of R837. The combination of these tumor-associated antigens and R837 effectively activated antitumor T cells. In vivo and in vitro studies demonstrated that RMmAGL immunoadjuvant NPs significantly suppressed the growth of primary tumors and suppressed metastases to prolong the survival of mice with metastatic lung tumors (Fig. 4) [221]. In spite of the R837 loading, RMmAGL provided excellent photothermal properties, resulting in a dramatic damage to the primary tumor tissue. Furthermore, mannose-induced macrophage polarization and R837-dependent DC maturation promoted this damage (Fig. 4C and D). As shown by H&E staining of the lungs (Fig. 4G) and the lung mass (Fig. 4H), tumor metastasis was also inhibited. These findings highlight the capacity of AuNPs to effectively activate the immune system and enhance antitumor activity.

Scientists have also demonstrated that the size and shape of Au NPs can affect the immunogenicity of vaccine compounds [232, 233]. Niikura and co-workers found that 40 nm spherical AuNPs coated with West Nile virus (Au NP-Es) elicited a high antibody titer, and was twice as effective as rod-shaped Au NP-Es in terms of antibody response [232]. It has also been shown that different shapes of Au NPs can activate different cytokine pathways. For instance, rod-shaped AuNPs significantly induced the production of interleukin (IL)-1β and IL-18, while both spherical AuNPs and cubic AuNPs greatly promoted the production of inflammatory cytokines (TNF-α, IL-6, IL-12) [232]. Despite the current positive results of Au NPs research in biomedical applications, there are still a number of issues that need to be addressed [215]. Their non-porous and non-biodegradable characteristics restrict their use for the time-release of small molecules [234]. In addition, although AuNPs are considered safe, the repeated use of AuNPs may result in bioaccumulation, which might have long term effects that are yet to be determined [166]. Further knowledge regarding the adjuvant efficacy of AuNPs is also required, as they are attractive candidates for vaccine carriers [235].

AuNPs, which have highly modifiable surfaces, biocompatibility, physiological stability, and ease of manipulation and manufacture, are considered promising candidates for vaccine development [216]. Due to their non-porous and non-biodegradable properties, they have limited applications [234]. However, despite these drawbacks, scientists are still utilizing AuNPs to boost the effectiveness of tumor immunotherapy. Future research will modify AuNP characteristics to overcome these weaknesses.

#### Sliver nanoparticles

**Fig. 5:**
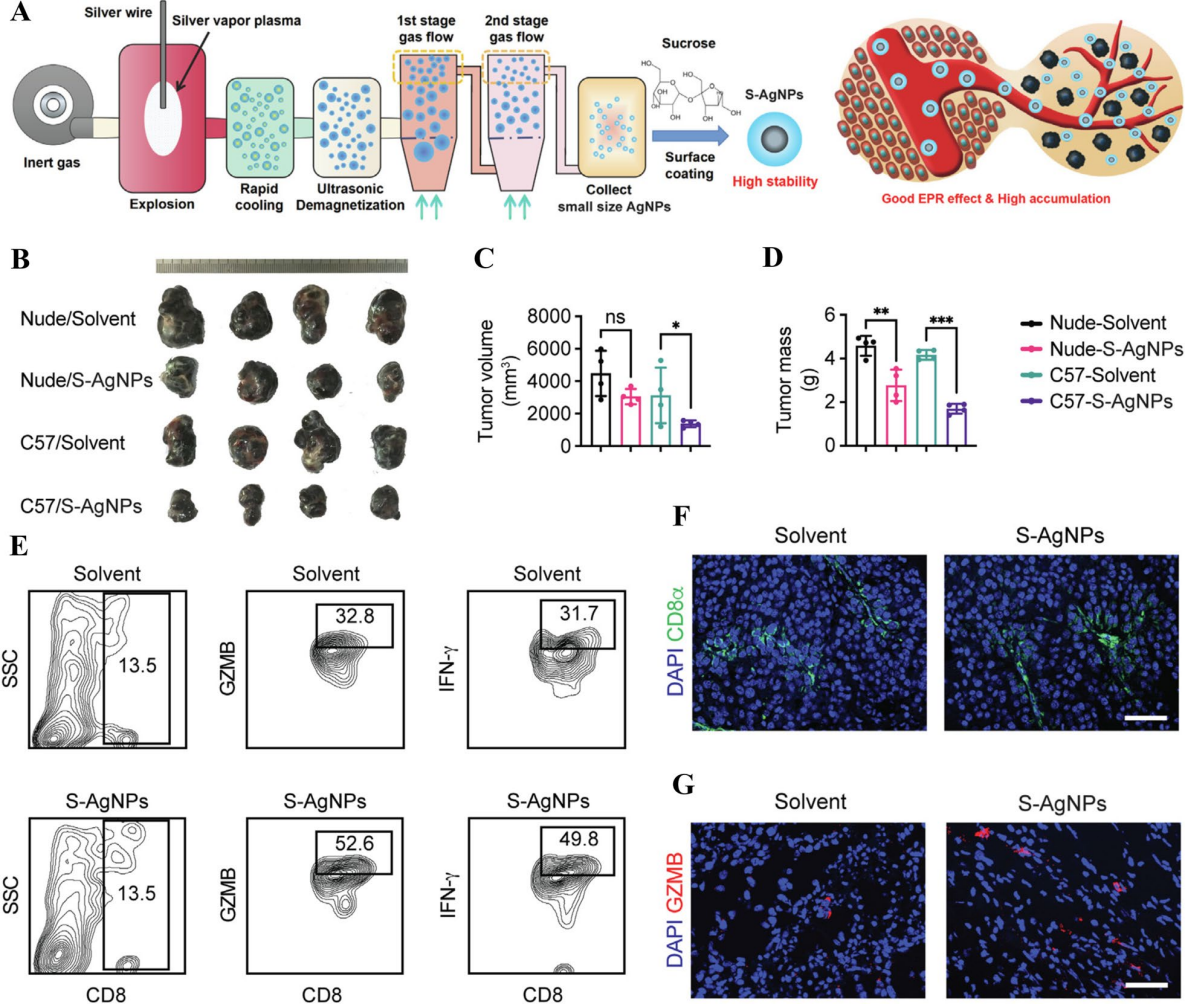
S-AgNP elicited a synergistic antitumor effect and induced CD8^+^ T cell activation in an immunocompetent mouse model. **A** The production of S-AgNPs was improved by redesigned evaporation–condensation protection systems. **B** An image of the tumor burden after S-AgNPs treatment in various B16-F10 tumor burden models. **C** Tumor volume was measured at the endpoint of the study in B16-F10 xenografts. **D** Tumor mass was measured at the endpoint of the study in B16-F10 xenografts. **E** A representative image of CD8^+^, CD8^+^GZMB^+^, and CD8^+^IFN-γ^+^ cells in CD45^+^ TILs collected from wild-type C57BL/6 B16-F10 xenografts treated with S-AgNPs. **F** and **G** S-AgNPs-treated C57BL/6 mice with B16-F10 xenografts are shown in images with IF staining for CD8 or GZMB. Adapted with permission from ref [251]. Copyright (2022) Journal of Colloid and Interface Science. S-AgNPs, AgNPs coated with sucrose; IFN-γ, Interferon-gamma; TEM, transmission electron microscopes; TILs, tumor infiltrating lymphocytes; GZMB, Granzyme B

As a potential multiplatform for enhancing cancer immunotherapy, sliver NPs (AgNPs) have recently received attention due to their unique properties [236–239]. They exhibited antimicrobial and anti-inflammatory characteristics [240–242]. Additionally, they are chemically stable and easy to synthesize [243–245]. Numerous studies have also demonstrated the antitumor properties of AgNPs [243, 244, 246–250]. The primary mechanism of action for AgNPs involves their ability to generate increased anticancer activity, induce DNA damage, and cause oxidative stress [239]. A recent study showed that the combination of honey with AgNPs exhibited the highest efficacy against hepatocellular carcinoma and colon cancer cells [243]. Similarly, Reddy et al*.* demonstrated the potent anticancer activity of AgNPs synthesized using *Perilla frutescens* leaf extract against human colon cancer and prostate adenocarcinoma cells [244]. Another investigation conducted by Mokhtar et al*.* involved synthesizing AgNPs using *Annona glabra* L. (AngTE) and *Annona squamosa* L. (AnsTE) through a biogenic route. They found that AnsTE and Ans-AgNPs were very effective at inducing apoptosis in human cervical cancer cells and ovary adenocarcinoma cells [246]. Kuang et al. reported that small sized Ag NPs exhibited potent antitumor activity, excellent druggability, and low systemic toxicity when combined with immune checkpoint blockade (ICB) therapy [251]. The results showed that these AgNPs induced cellular apoptosis and promoted the infiltration and activity of cytotoxic CD8^+^ T cells, leading to inhibited tumor cell proliferation (Fig. 5). Flow cytometric analysis demonstrated that the S-AgNP-treated groups had considerably higher tumor-infiltrating CD8^+^ T cell activity (GZMB^+^ or IFN-γ^+^) (Fig. 5E). In the S-AgNP-treated group, immunofluorescence (IF) staining clearly showed an increase in CD8^+^GZMB^+^T cells in the tumor area (Fig. 5F and G). These findings suggest that small sized AgNPs could serve as a potential adjuvant for immunotherapy, offering a novel clinical treatment strategy by combining small sized AgNPs with pair programmed cell death protein 1 (PD-1) mAbs in the future (Fig. 5) [251].

Furthermore, there have been a report on the conjugation of AgNPs with anticancer agent [252]. For example, Saeidi et al. evaluated the cytotoxic effects of greenly synthesized AgNPs (GS-AgNPs) combined with doxorubicin on cancerous cells (MCF7) and normal heart cells (H9c2) [252]. Coffee extracts were used as a reducing and stabilizing agent for the green synthesis of AgNPs. In comparison with chemically synthesized NPs, GS-AgNPs were more biocompatible with normal cells and more toxic towards cancerous cells [252]. Zou et al*.* modified AgNPs with the organic drug Paclitaxel (PTX) and evaluated their effect on adenocarcinomic human alveolar basal epithelial cells (A549 Cells) [253]. The results showed that Ag@PTX significantly reduced the viability of A549 cells. Moreover, Ag@PTX enhanced the anti-cancer activity of A549 cells by activating ROS-mediated p53 and AKT pathways. In nude mice xenograft models, Ag@PTX effectively suppressed tumor growth, indicating its potential as a highly efficient solution for achieving anti-cancer synergism in humans [253]. In a recent research, Muhammad et al. also investigated the effect of AgNPs functionalized PTX nanocrystals and polydopamine (PDA) on human cancer cells [254]. They initially prepared PTX nanocrystals as templates and then coated them with PDA. The PDA layer facilitated the in-situ production and deposition of AgNPs, as well as the grafting of tumor-targeting peptide NR1 (RGDARF). The functionalized NPs exhibited significantly enhanced their uptake efficiency in cells, demonstrated strong anti-cancer activity in vitro, and showed anti-migratory properties against a variety of cancer cells [254]. Furthermore, these nanocrystals showed strong potential for inducing apoptosis, characterized by membrane lysis, nuclear damage, mitochondrial dysfunction, excess ROS release, and double-stranded DNA damage [254]. Additionally, AgNPs trigger an inflammatory reaction cascade that involves macrophages, neutrophils, and helper T cells. The AgNPs then induce the production of several different kinds of cytokines [255]. A number of researchers have examined the immunological adjuvant effectiveness of AgNPs both in vitro and in vivo [256, 257]. According to Kuang et al., AgNPs have a potent adjuvant effect [251]. They developed small size Ag NPs coated with sucrose (S-AgNPs) as potent adjuvants to study combination therapies. S-AgNPs' anticancer effects were examined in vitro and in comparison in melanoma-affected immunodeficient and immunocompetent mice. Their research revealed that S-AgNPs had strong anticancer effects, good druggability, and minimal systemic toxicity. Mechanistically, they demonstrated that S-AgNPs stimulate cytotoxic CD8^+^ T cell infiltration and activation while inhibiting tumor cell proliferation by causing cellular apoptosis [251].

While AgNPs offer numerous advantages, they also have some drawbacks. AgNPs were reported to have toxicity to most human cell lines [258]. However, Bae et al. claimed AgNPs only triggered inflammatory responses (IL-2, IL-17A, IL-17F, MIP1β, TNFα, and IFNγ) in human peripheral blood mononuclear cells (hPBMCs) under the specific conditions examined in the study, rather than causing cytotoxicity [259]. The potential negative effects of AgNPs on mammalian cells remain a subject of debate and necessitate further investigation.

Over the last decades, significant progress has been made in the field of AgNPs-based cancer vaccines. It is easy to synthesize AgNPs and they are chemically stable [244]. Additionally, numerous studies have shown that AgNPs have anticancer effects [252–254]. Although they may have toxicity drawbacks, this remains a topic of debate.

#### Silica nanoparticles

**Fig. 6:**
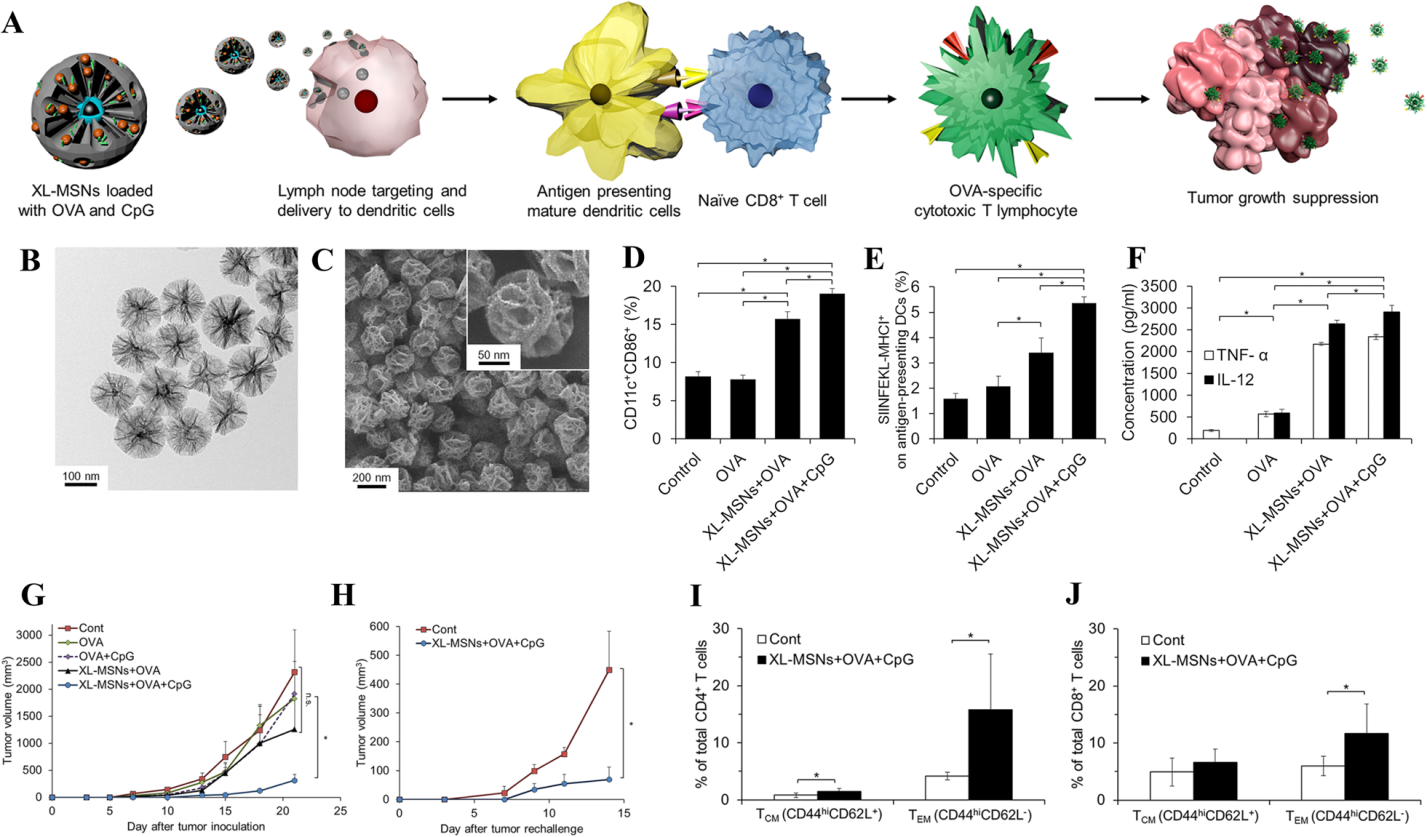
Co-delivery of Toll-like receptor 9 agonist and protein antigen by extra-large pore mesoporous silica nanoparticles for enhancing cancer vaccine efficacy. **A** This schematic illustration illustrates how extra-large mesoporous silica NPs are used to induce antigen specific CTLs. **B** and **C** TEM and SEM images of silica NPs. **D** A CD11c^+^CD86^+^ BMDC activation. **E** Flow cytometry analysis of BMDCs presenting antigenic SIINFEKL peptides on their MHC molecules. **F** Measurement of BMDCs' secreted TNF-α and IL-12 by ELISA. **G** The growth of the tumor after tumor injection till day 21. **H** 15 days after an inoculation of OVA-KO cells with 1 × 10^6^ of XL-MSNs coloaded with CpG and OVA, tumor-free mice were rechallenged with 1 × 10^6^ of B16-OVA cells. **I** and **J** The number of CD4 and CD8 memory T cells in the spleens of vaccinated mice was measured by flow cytometry. Adapted with permission from ref [266]. Copyright (2018) ACS Central Science. CTLs, cytotoxic T lymphocytes; TEM, transmission electron microscopes; SEM, scanning electron microscope; BMDC, bone marrow-derived dendritic cells; TNF, tumor necrosis factor; ELISA, enzyme-linked immunoassay; XL-MSNs, extra-large pore mesoporous silica nanoparticles; OVA, ovalbumin; IL, interleukin

Silica NPs, specifically MSNs, have been considered for vaccine carrier development, alongside other inorganic NPs [260–263]. Despite the significant promise demonstrated by polymeric and lipid NPs as vaccine delivery systems, these NPs suffer from instability and rapid degradation during interstitial transit [264]. The polymer matrix hydrolyzes, resulting in the release of encapsulated or adsorbed molecules (such as antigens or drugs) within hours of administration [264]. This presents a major obstacle in vaccine development. In recent years, silica NPs have drawn considerable attention as a potential solution to antigen leakage and NP instability [260]. Silica NPs offer advantages such as easy control over size, shape, and structure. Additionally, silica exhibits excellent chemical stability, biocompatibility, and can be easily modified through surface functionalization [212]. These characteristics make silica NPs highly promising vehicles for protein, gene and drug delivery [260]. Silica NPs could reinforce the immune response both as a vaccine adjuvant and delivery vehicles [261, 265]. Toda et al*.* found that smaller-sized silica NPs (30 nm) exerted greater adjuvant effects and promoted stronger T helper (Th)1, Th2, and Th17 immunity compared to larger-sized silica NPs (100 nm and 1000 nm) [265]. However, a recent study by Shin and coworkers have shown that large-sized silica NPs (∼350 nm) also induced specific antigen-specific immune responses. Large silica NPs facilitated the production of an antigen supply depot at the injection site, resulting in robust immune responses, including cellular and humoral immunity against tumors [68]. Similarly, Cha et al*.* revealed that large silica NPs (100–200 nm) delivered with TLR 9 and OVA antigen could enhance the effectiveness of cancer vaccines (Fig. 6) [266]. In vitro studies showed improved DC activation, antigen presentation, and cytokine production. Animal studies demonstrated successful antigen transport and TLR9 agonist delivery to draining lymph nodes, triggering antigen-specific CTLs, and inhibiting tumor growth after vaccination [266]. Compared to all other groups, extra-large pore mesoporous silica NPs (XL-MSNs) coloaded with OVA and CpG significantly inhibited tumor growth (Fig. 6G). Furthermore, vaccinated mice showed a significant increase in memory T cell numbers compared non-vaccinated counterparts [266]. One week following vaccination, the population of memory T cells was examined by examining the subsets of effector memory T cells (TEM) and central memory T cells (TCM) (Fig. 6I and J). Silica NPs have also demonstrated the ability to enhance mucosal and systemic immunity and can serve as carriers for oral vaccines targeting various infectious diseases (such as hepatitis and influenza) [267]. While silica NPs hold promise as vaccine carriers, further research is needed to better understand their adjuvant efficacy.

Briefly, silica is very biocompatible, has good chemical stability, and is easily changed by surface functionalization. Another advantage of silica NPs is that their size, shape, and structure are easily controllable [212]. Moreover, they may strengthen the immune system when used as vaccine adjuvants and delivery systems [268]. Therefore, silica NPs hold great potential in cancer immunotherapy.

#### Calcium phosphate nanoparticles

In recent years, there has been a rapid increase in the utilization of calcium phosphate NPs (CaP NPs) in cancer vaccine research [269–275]. CaP NPs exhibit great potential as nanocarriers for the treatment of various diseases, such as infectious diseases and cancer, owing to their biocompatibility and physicochemical properties [276–280]. These NPs are non-toxic, biodegradable, cost-effective, pH-sensitive, and can be synthesized in different shapes, sizes, and surface charges [210, 281]. In addition, CaP NPs can be functionalized with a variety of molecular adjuvants to enhance immune cell targeting and vaccine efficacy [282, 283]. They can also be modified to carry peptides, proteins, and DNA vaccine cargo by adding hydrophilic or hydrophobic molecules [284]. Additionally, CaP-NPs show promise as gene therapy agents, making them well-suited for application in cancer immunotherapy [285, 286]. As a result, CaP NPs hold significant potential as universal adjuvants for inducing both humoral and cellular immunity [287, 288].

**Fig. 7:**
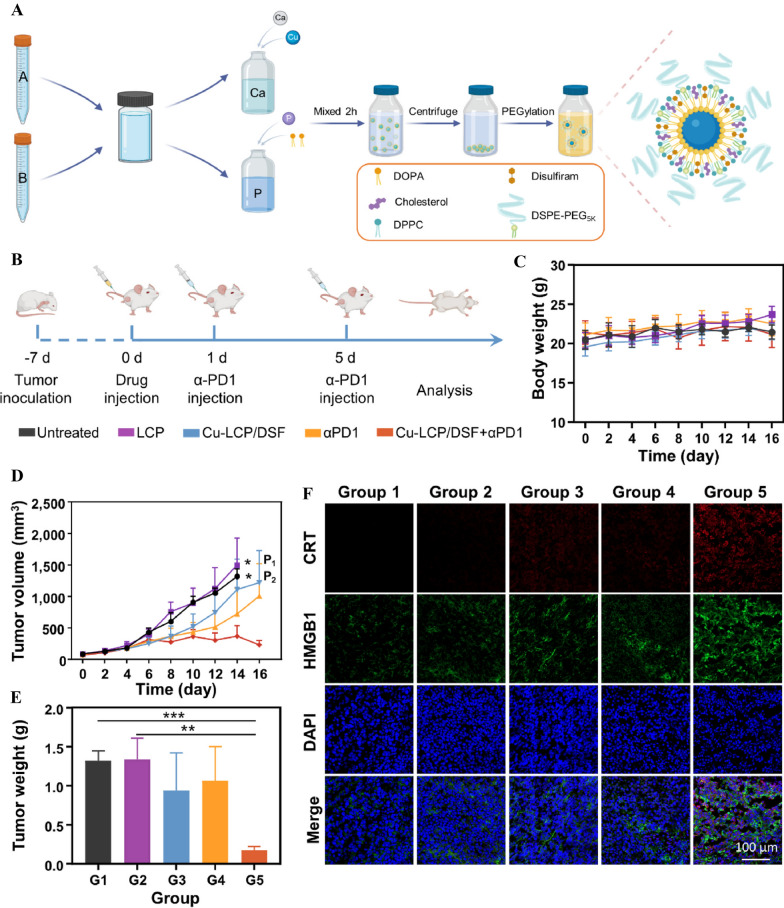
CaP NPs loaded with disulfiram for improved cancer immunotherapy. **A** An illustration of the fabrication procedure for Cu-LCP/DSF NPs. The solution in tube A is cyclohexane/Igepal CO-520; the solution in tube B is cyclohexane/Triton-X 100/hexanol. **B** A schematic illustrating the combined Cu-LCP/DSF NPs and anti-PD-1 treatment schedule for the mouse model CT26. **C** Mice's weight after treatment. **D** CT26 tumor growth curves after different treatments in tumor-bearing mice. **E** Different treatments' tumor inhibitory rates based on tumor volume. **F** Images of different groups of mice with tumor slices stained with CRT and HMGB1. Adapted with permission from ref [274]. Copyright (2022) Biomaterials. DOPA, 1, 2-dioleoyl-sn-glycero-3-phosphate (sodium salt); DPPC, 1,2-dipalmitoyl-sn-glycero-3-phosphatidylcholine; DSPE-PEG_5k_, 1,2-distearoyl-snglycero-3-phosphoethanolamine-N- (methoxy (polyethylene glycol)-5000); LCP, lipid-coated calcium phosphate; DSF, Disulfiram; PD, programmed cell death; HMGB1, high mobility group box protein B1; CRT, calreticulin

In 2019, Heße and colleagues employed CaP NPs functionalized with CpG and tumor antigen to induce an immune response against colorectal cancer [289]. The therapeutic vaccination with CaP cancer vaccine was found to increase cytotoxic CD8^+^ T cells in tumors in an interferon-dependent manner. Additionally, combining CaP NPs vaccine with PD-L1 immune checkpoint blockers significantly increased CD8^+^ T cells infiltration in tumors and facilitated their eradication [289]. In another study, Wang et al. utilized a lipid-coated calcium phosphate (LCP) mRNA vaccination encoding tyrosinase-related protein 2 (TRP2) in a C57BL/6 mouse model of B16F10 melanoma. The vaccination elicited a robust antigen-specific cytotoxic T cell response as well as a humoral immune response, effectively inhibiting the growth of melanomas [277]. Moreover, Liu et al. employed CaP NPs coated with lipids as a carrier to deliver the BRAF^V600E^ peptide (mutant melanoma) to C57BL6 mice with BRAF-mutant melanoma [290]. The BRAF peptide vaccination induced potent cytotoxic T cell responses, inhibited tumor growth, and enhanced infiltration of CTLs by remodeling immunosuppressive modules within the tumor microenvironment [290]. A recent investigation by Sun et al. demonstrated that mannose-functionalized CaP NPs efficiently delivered a DNA vaccine and promoted antitumor immunity. They demonstrated that the immunization with mannose-modified and bisphosphonate (BP)-stabilized CaP NPs significantly inhibited the growth of E.G7 cells expressing OVA antigen in the C57BL/6 J mice [291]. To enhance the weak immunogenicity of the vaccine, the same group later utilized adenosine triphosphate (ATP) as both a stabilizing agent for CaP and an immunological adjuvant to the DNA vaccine. The mice given the ACP-DNA vaccine displayed increased antigen-specific antibodies and a greater suppression of tumor growth [292]. Moreover, CaP NPs can be combined with drugs to enhance cancer immunotherapy. Li et al. developed pH-responsive lipid-coated CaP NPs (LCP NPs) co-loaded with Cu^2+^ and disulfiram (DSF) (Fig. 7) [274]. As a result of intravenous injection, those NPs accumulated in tumors due to their long blood half-life and were degraded in the acidic tumor microenvironment, releasing Cu^2+^ and DSF to produce the cytotoxic metabolite DTC-Copper complex, bis(diethyldithiocarbamate)–copper (CuET). CuET could efficiently induce immunogenic cell death in cancer cells, modulating the immunosuppressive microenvironment of the tumor (Fig. 7) [274]. It was found that Cu-LCP/DSF NPs combined with anti-programmed cell death protein 1 (anti-PD-1) therapy showed excellent tumor regression (Fig. 7E). In comparison to the control group, calreticulin (CRT) and high mobility group box protein B1(HMGB1) expression were significantly higher in tumor tissue after combined treatment with Cu-LCP/DSF NPs and anti-PD-1 (Fig. 7F). It has recently been found that activating the cyclic guanosine monophosphate–adenosine monophosphate synthase-stimulator of interferon genes (cGAS-STING) pathway could enhance natural immunity and increase lymphocyte infiltration into tumor microenvironments [293]. Xiao et al. prepared hydroxyapatite NPs that were co-loaded with curcumin and L-oxaliplatin (Cur/L-OHP@HAP NPs). The formulated Cur/L-OHP@HAP NPs were evaluated both in vitro and in vivo for anti-tumor properties and immune activation [293]. They found that HAP promotes the release of intracellular Ca^2+^ stores and curcumin inhibits Ca^2+^ efflux, resulting in intracellular Ca^2+^ overload and release of mitochondrial DNA. Both nuclear DNA and mitochondrial DNA damage significantly increased the cGAS-STING pathway's activation, which in turn led to the recruitment of immune cells to the TME and the activation of natural immunity. Thus, with the use of Cur/L-OHP@HAP NPs, cancer immunotherapy may be greatly improved [293]. These studies demonstrate the effectiveness of CaP NPs as a nano-delivery system and nano-adjuvant for cancer vaccines. However, limitations such as limited antigen loading capacity and rapid NP aggregation remain significant challenges [139]. Despite these challenges, the use of CaP nanomaterials still holds great potential in cancer applications.

CaP NPs are effective vaccine adjuvants and delivery vehicles [284]. As well as being non-toxic, biodegradable, and cost-effective, CaP NPs can alter their physical characteristics such as size, shape, and surface charge by modifying pH [281]. Furthermore, CaP NPs can be functionalized with molecular adjuvants that enhance active immune cell targeting [283]. Additionally, CaP NPs can be modified with both hydrophilic and hydrophobic molecules to transport peptide, protein, or DNA vaccine cargo, making them a universal adjuvant [284]. Despite CaP NPs' potential for vaccine development, their clinical use has been limited by a number of factors. CaP NPs are usually low in antigen-loading capacity and aggregate quickly [294]. Although there are several obstacles in the way of using NPs as vaccine carriers, CaP NPs will offer a potential new platform and carrier for the creation of successful cancer vaccines.

In conclusion, the application of inorganic NPs in cancer vaccine applications offers a promising avenue for the development of innovative and effective therapeutic strategies. AuNPs are easily functionalized, providing a versatile platform for antigen delivery and immune system regulation. Similarly, AgNPs exhibit excellent biocompatibility and adjuvant properties, contributing to their widespread adoption in cancer vaccine formulations. Recognized for their stability and tunable surface properties, silica NPs provide an attractive platform for antigen encapsulation and controlled release. CaP NPs are biocompatible and biodegradable and play a crucial role in enhancing the stability of antigen and promoting the activation of APCs.

The diversity of inorganic NPs underscores the adaptability of nanotechnology in tailoring vaccine formulations for cancer immunotherapy. However, it is important to acknowledge the current challenges associated with inorganic NPs, such as their potential toxicity and variability in immune responses. As research in the field progresses, addressing these challenges will be key to the successful transition of inorganic NP-based cancer vaccines from the laboratory to clinical application.

### Virus-like particles

In recent years, there has been a significant surge of interest in virus-like particles (VLPs) within the field of biomedical research [60, 295–300]. VLPs possess a viral structure but lack of viral genetic material, making them safe and non-replicative [60, 301]. These VLPs are formed by the self-assembly of biocompatible capsid proteins, effectively eliminating any infectious nucleic acids [302]. The numerous advantages associated with VLPs include their capacity for large drug loading, immunogenicity, adjuvant activity, and their ability to facilitate non-toxic and targeted delivery [303, 304]. Furthermore, since they possess an innate viral structure, VLPs do not infect the immune system [302]. They can also be fabricated in a range of sizes, spanning from 20 to 800 nm, and through a variety of production methods [305]. It is important to note that VLPs may cause side effects, such as injection site pain and swelling [302]. Given these factors, it is unsurprising that VLPs have become an attractive platform for vaccine design over the past two decades [69, 301, 306–310].

The presence of squalene oil-in-water adjuvant (MF59) with chimeric VLPs, which presented tumor-associated mucin 1 (MUC1) epitopes, led to the induction of high levels of specific IgG antibodies [306]. Moreover, Li et al. developed an efficient VLP-based nanoplatform for antigen delivery to elicit an effective CTL reaction [311]. They utilized OVA B and T epitopes as model antigens, loading peptide antigens onto P22-derived VLP surfaces. Their study in mouse tumor model revealed that VLP-OVAT effectively suppressed tumor growth through the promotion of CD4^+^, CD8^+^, and effector memory T cells (T_EM_ cells) and the reduction of myeloid-derived suppressor cells (MDSCs) within tumor-infiltrating lymphocytes and spleenocytes [311].

**Fig. 8:**
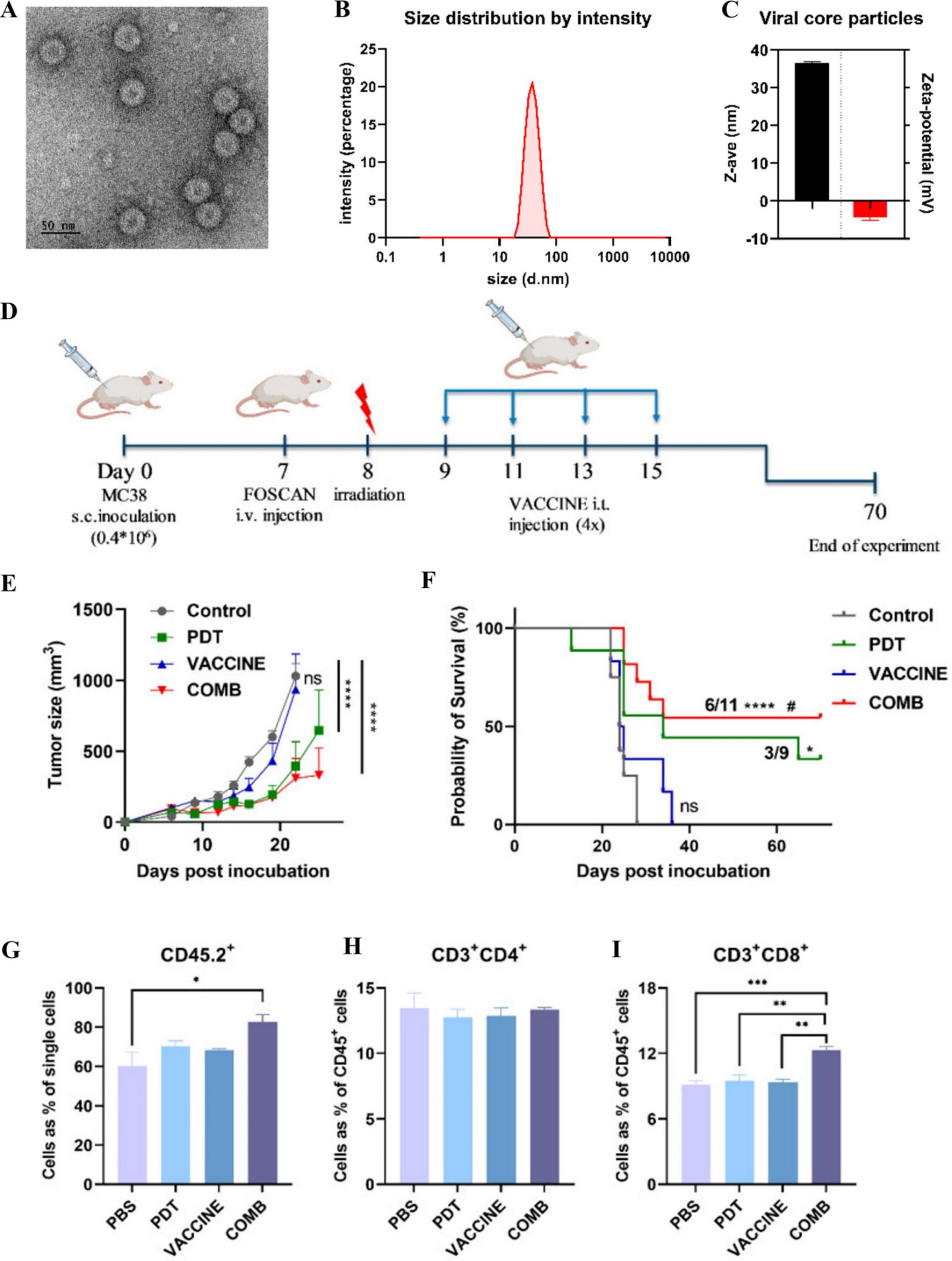
The Hepatitis B Core (HBc) VLPs in combination with photodynamic therapy to prime anticancer immunity. **A** TEM images of HBc VLPs. **B** Particle size histogram for the viral core. **C** HBc VLP size average (z-ave) and zeta potential. **D** Experimental design of the MC-38 tumor model. **E** After treatment, the average size of MC-38 tumors in mice. **F** A plot of Kaplan–Meier survival curves shows survival times for subgroups. Following the second vaccination, spleens were collected and processed, followed by flow cytometry analysis to analyze immune cell populations in mice from different subgroups. (**G**, **H** and **I**) CD45.2^+^, CD4^+^, CD8^+^ T cell population. Adapted with permission from ref [314]. Copyright (2022) Cancers. HBc VLPs, Hepatitis B Core Virus-like Particles; PDT, Photodynamic therapy; Vaccine, meta-tetrahydroxy-phenylchlorin (mTHPC, trade name FOSCAN)-based PDT; COMB, PDT in combination with HBc VLPs

Additionally, VLPs can be used to deliver multiple antigens to improve cancer immunotherapy. Jiménez-Chávez Á et al. created and assessed the therapeutic efficacy of VLPs presenting the VP2 protein of the human parvovirus B19, along with several neoepitopes (Tmtc2, Gprc5a, Qars). Compared to treatment with wild-type VLPs, treatment with multi-epitope VLPs significantly delayed tumor growth and reduced the number of lung macrometastasis [312]. Two years later, Campbell et al. reported that the antitumor immune response is enhanced by delivering two tumor antigens, Survivin and Mucin-1 to a VLP-based BC vaccine (CpG as a vaccine adjuvant). They found that two tumor antigens are simultaneously delivered from the VLPs, inducing a stronger immunity against the tumor compared to delivering a single antigen [313]. Furthermore, a combined therapy strategy holds promise for cancer treatment. In 2022, Hao et al. utilized Hepatitis B Core (HBc) VLPs in combination with photodynamic therapy (PDT) to prime anticancer immunity (Fig. 8) [314]. PDT is a local treatment that employs photosensitizers, which is a drug that becomes activated to produce a form of oxygen to kills nearby cells when exposed to light. By combining PDT with immune agents, the anti-cancer efficiency can be further enhanced. To improve the immune response to PDT, the researchers incorporated a viral vaccine using HBc VLPs. A significant delay in tumor growth was observed for three weeks following meta-tetrahydroxy-phenylchlorin (mTHPC, trade name FOSCAN)-PDT treatment and PDT in combination with HBc VLPs (COMB) therapy (Fig. 8E). The COMB treatment had a higher survival rate (55%) than PDT alone (33%) (Fig. 8F). Their study in a murine colorectal tumor model MC-38 demonstrated that the combination therapy enhanced innate and humoral immune responses, prolonged survival, and long-term memory capacity (Fig. 8) [314].

More recently, Van et al*.* demonstrated the potential of a naturally occurring encapsulin delivered from *Thermotoga maritima* as a functional delivery system for breast cancer cells [296]. By using a single plasmid in *Escherichia coli*, they co-expressed an engineered flavin-binding protein mini-singlet oxygen generator (MiniSOG) and an encapsulin-Designed Ankyrin repeat protein (DARPin) 9.29 fusion protein, allowing for the generation of drug delivery systems in a single step. The DARPin9.29 used in this investigation particularly binds human epidermal growth factor receptor on breast cancer cells. These formulated nanocompartments exhibited specific targeting towards human epidermal growth factor receptor 2 (HER2) positive breast cancer cells and caused apoptosis [296].

As a result, there are many advantages to VLPs, including substantial drug loading capacity, immunogenicity, adjuvant activity, and a potential for tailored and non-toxic delivery [303]. Additionally, since VLPs are innately viral, they do not affect the immune system [302]. They can also be made in a variety of sizes [69]. According to the investigations above, it is important to realize that, although there are still some limitations to the VLP platform, recent advances in the field, together with the ability to engineer VLPs and use appropriate adjuvants, offer more opportunities for designing and manufacturing more effective VLPs for cancer prevention and treatment [69, 315].

### Immunostimulatin complexes

Immunostimulatin complexes (ISCOMs) are a popular form of nanovaccines in vaccine research [113, 316–318]. ISCOMs are cage-like particles, typically 40–50 nm, that spontaneously form from phospholipids, cholesterol, saponin, and protein antigens [319–321]. However, one limitation of ISCOMs formulations is that they require amphipathic proteins, which restricts the types of antigens that can be incorporated [319]. Alternatively, ISCOMs can be formulated without antigens, resulting in a structure known as ISCOMATRIX™, which closely resembles that of ISCOMs [322]. ISCOMATRIX™ can be used to formulate antigens to create ISCOMATRIX™ vaccines, offering similar antigen presentation and immunomodulatory properties without the limitation of being restricted to hydrophobic membrane proteins [319]. Due to these properties and their acceptable safety profile, ISCOMs and ISCOMATRIX™ serve as suitable adjuvants for cancer vaccines [321].

Numerous studies have provided evidence that ISCOMs and ISCOMATRIX™ vaccines can induce robust immunological responses to a wide range of antigens in many animal models and clinical trials [113, 323–328]. Co-administration of antigens with ISCOMs or ISCOMATRIX™, trigger innate responses, followed by antibody responses and effector CD4^+^ and CD8^+^ T-cell responses [329–331]. For instance, Silva et al. conducted research to evaluate the efficacy of the combination of ISCOMATRIX™ and TLR agonists in several solid tumors [332]. The results showed that the co-administration of polyinosinic-polycytidylic acid (PolyI:C) and CpG with ISCOMATRIX™ vaccines significantly reduced tumor growth in all tested tumor models (melanoma, pancreatic cancer, and prostate cancer). The combination of these vaccines induced a robust response in CD8^+^ T cells. In another study by the same group, aimed at identifying a vaccine with therapeutic protection against cancers with poor immunogenicity, an ISCOMATRIX™ prostate cancer vaccine was developed using the tumor antigen prostatic acid phosphatase (mPAP), the TLR3 agonist PolyI:C, and the immunostimulatory cytokine FMS-like tyrosine kinase 3 ligand (Flt3L) [333]. The study found that 60% of animals treated with ISCOMATRIX™-mPAP-Poly I:C-Flt3L in a therapeutic prime-boost regimen showed complete tumor remission, and these tumor-free animals were protected from recurrence upon reexposure. The vaccine also showed effectiveness in two additional cancer models, B16-OVA melanoma and E-myc-GFP-OVA lymphoma [333]. HCA587 is an antigen present in a number of malignancies has and possesses unique immunological properties, making it a promising target for immunotherapy [334]. Chen et al. reported that the HCA587 protein formulated with CpG and ISCOM induced a strong cellular and humoral immune response, as evidenced by high levels of HCA587-specific antibodies and CD4^+^ T cells. Vaccination with HCA587 provided both prophylactic and therapeutic protection against HCA587-expressing B16 melanoma [334]. In a similar study by Yang et al., the cancer vaccine encoded with HCA587, CpG and ISCOM was evaluated to assess its immunogenicity. Vaccination with the HCA587 protein vaccine induced significant immune responses, resulting in slowed tumor growth and improved survival in mice. The vaccination also increased the proportion of CD4^+^ T cells expressing granzyme B and IFN-γ in tumor tissues, suggesting their contribution to the antitumor effect [335]. In another investigation, Klein and co-workers demonstrated that a low-dose of cyclophosphamide enhanced the CD4^+^ T cell response to the NY-ESO-1/ISCOMATRIX™ vaccine in patients with advanced melanoma in clinical trial [324]. Based on these findings, ISCOMs and ISCOMATRIX™ have great potential as adjuvants in the development of cancer vaccines [320].

As a whole, nanovaccines such as ISCOMs are popular in vaccine research [316, 333]. They have high stability, less toxicity, and strong adjuvant properties [336, 337]. In addition, they do not have hemolytic activity. ISCOMs against certain antigens have been shown to elicit both humoral and cellular immune responses, such as CD4^+^ helper T cells and CD8^+^ cytotoxic T cells [329]. However, the ISCOMs method is limited to integrating hydrophobic membrane proteins, which is one of its drawbacks. Nevertheless, ISCOMs and ISCOMATRIX™ will have great potential for cancer vaccines when the scientists resolve the issues [338].

The advantages and drawbacks of the above-mentioned NPs for nanovaccines applications are summarized in Table 1. These NPs offer benefits such as improved antigen stability, targeted delivery, and long-term release, achieved through encapsulation or surface modification [136]. They can be surface-engineered with peptides, proteins, polymers, cell-penetrating peptides, and other targeting ligands due to their large surface area-to-volume ratios, controllable size and shape, and a variety of surface charges [113]. However, drawbacks include unfavorable interactions with the reticuloendothelial system (RES) and limited colloidal stability in physiological settings because of protein corona formation [339]. In recent years, biomimetic NPs have emerged as innovative natural mimicking biosystems that are useful for cancer immunotherapy [340, 341]. The biomimetic NPs exhibit improved colloidal stability and avoid unwanted interactions with immune cells like the RES, while prolonging circulation in the blood [342].

**Table 1:** Advantages and disadvantages of different types of nanovaccines

Type	Advantages	Disadvantages	Refs
Polymeric NPs	• Biodegradable and biocompatible • Water-soluble, non-toxic • Inexpensive • Easy to manufacture • Stable	• Short half-life • Low encapsulation efficiency • Insufficient drug loading capacity • Weak solubility	[147, 150, 151, 175]
Lipid NPs	• Good biocompatibility • Be able to enclose various agents • Have versatility, and plasticity • Low toxicity • Increased drug dosages • Synthesized in a wide range of sizes, compositions, and lipid loads	• Not Stable • Lipid dispersion gelation • Hydrophilic drug loading capacity is limited • Low encapsulation efficiency	[102, 196, 353–355]
Gold NPs	• Biocompatible, • Physiologically stable • Easy to manipulate and manufacture • NP surface can be modified with diverse molecules • Lower systemic toxicity • Higher tumor accumulation • Faster kidney clearance • Tunable chemical reactivities	• Non-porous • Non-biodegradable • Bioaccumulation	[211, 215, 356–358]
Silver NPs	• Anticancer activity • Antibacterial properties • Anti-inflammatory • Chemical stability • Ease of synthesis	• Toxicity to mammalian cells	[241, 243, 258, 359]
Silica NPs	• Excellent chemical stability • Good biocompatibility • Facile surface modification • Easy to control the size, shape, and structure • High porosity • Self‐adjuvanticity	• Difficult in preparation of well-ordered • Scattered size distribution • Formation of stable-colloidal suspensions	[106, 212, 261, 268, 360]
Calcium phosphate NPs	• Safety, biocompatibility and stability • pH-dependent solubility • Surface modification • High adjuvanticity • High biodegradability • Greater affinity to biological materials	• Low antigen loading capacity • Rapid aggregation	[276, 294]
Virus-like particles	• Large drug-loading • Antigenicity, safe • Adjuvant activity • Without causing infections • Targeted delivery • Considerable safety	• Pain • Swelling after injection • Polydispersed particle size • Limited encapsidation	[302–304]
Immunostimulating complexes	• More immunogenic • High stability • Less toxicity • Strong adjuvant properties • Do not have hemolytic activity	• Limits the binding of neutral or negatively charged hydrophilic antigens • Exert no depot release profile	[113, 319, 320, 331, 361]

The wide application of NPs in cancer vaccine development spans a variety of therapeutic modalities, including adoptive cell therapy, artificial antigen presentation, and biomimetic immune-activation. Adoptive cell therapy (ACT) involving tumor-infiltrating lymphocytes (TILs) or genetically modified T cells expressing novel T cell receptors (TCR) or chimeric antigen receptors (CAR) is an approach that modifies the immune system to recognize tumor cells and thus function as anti-tumor effects [343]. In adoptive cell therapy, NPs play a key role in enhancing the delivery of therapeutic agents to immune cells and promoting tumor targeting and cytotoxicity [344]. Polymer NPs, known for their biocompatibility and tunable properties, provide a flexible platform for adoptive cell therapy applications. For instance, a nanostructured polyethylene glycol (PEG) hydrogel platform has been developed to stimulate T cells before adoptive transfer, improving ex vivo expansion of antigen-specific T cells [345]. Furthermore, polymeric nanocarriers that encapsulate mRNA have also been explored to transiently deliver mRNA to antigen-specific T cells prior to adoptive transfer [346].

Currently, the artificial APC (aAPC) technique stands as a cell-based therapeutic approach that can significantly enhance the immune response in comparison to TCL-based vaccinations [347]. NPs act as an effective carrier of antigen and promote the effective presentation of antigen to immune cells [348]. An investigation by Song revealed that the combination of TCL-poly (lactic-co-glycolic acid)-PEI (TPP) tumor nanovaccines and aAPCs induced a greater immune response and achieved better antitumor results than individual therapies. This combined therapy increased proliferation activities, inhibited regulatory T cells, promoted inflammatory cytokine production, and reduced inhibitory cytokine production [347]. Lipid NPs, due to their lipid bilayer structure, are well suited to simulate cell membranes and enhance antigen presentation and subsequent immune response [349].

Biomimetic immune activation utilizes the ability of NPs to replicate natural processes within the immune system. In this case, gold NPs, with their unique physicochemical properties, can regulate the activation of immune cells and the release of cytokines [350]. Silica NPs, recognized for their stability and tunable surface properties, have found use in biomimetic immune activation to provide controlled release of immunomodulators [351]. The biocompatibility and biodegradability of calcium phosphate NPs contribute to their efficient formulation of safe and effective nanovaccines [352]. However, challenges such as potential toxicity and variability in immune responses remain critical considerations, necessitating ongoing research to successfully translate NP-based cancer vaccines from laboratory settings to clinical practice.

## Challenges and future directions

The above mentioned nanovaccines have demonstrated the ability to elicit immune responses in both the cellular and humoral systems [113, 362]. Nevertheless, alongside the potential benefits of employing NP vehicles in forthcoming vaccination strategies, prudent consideration of certain limitations remains pivotal [363]. Overcoming these challenges and expediting the clinical translation of an expanded array of nanovaccines necessitates continued innovation and advancements in the field.

Several challenges need to be addressed prior to the successful translation of nanovaccines into clinical practice [20, 135, 136]. Foremost, upholding a consistent and reproducible manufacturing process is imperative to ensure the efficacy and uniformity of engineered NPs, encompassing their characterization and performance [356, 364]. Strategic optimization of NP properties, such as size and ligands, assumes pivotal significance in advancing NP integration within clinical paradigms. Moreover, the stability of nanomaterials under physiological conditions is a significant concern for medical application [365]. The biodegradability and solubility of materials (particularly inorganic nanocarriers), present additional complexities [77, 366]. Further, prudent consideration must be given to the potential formation of bio-coronas encasing NP surfaces, urging the formulation of protocols or strategies to mitigate this phenomenon in future nanovaccine developments [356]. Additionally, the comprehensive exploration of distinct NPs that intrinsically enhance immunopotentiation effects within cancer vaccines have yet to be fully elucidated and should be investigated on a fundamental level [14]. A profound comprehension of the interactions between NPs and the immune system, coupled with their in vivo distribution, stands as a fundamental prerequisite for the design of more effective nanoformulations [356]. The potential lies in harnessing single-cell sequencing technology to dissect how NPs interact with specific APCs [14]. Besides, the aspect of safety necessitates thoughtful contemplation in the context of nanovaccine deployment [339]. Thorough studies on NP toxicity are necessary, given the potential alterations in physicochemical properties of NPs subsequent to interactions with other biological substances within the body [364].

Although cancer vaccines showed great promise in preclinical studies, most of them failed to provide clinical benefits to patients, especially those with advanced cancer [367]. Poor clinical outcomes can result from various factors, including high levels of tumor heterogeneity, low immunity, poor solid tumor infiltration, immune tolerance, lack of appropriate tumor antigens and regulatory hurdles [367, 368]. Treatment optimization and patient outcome prediction are challenging due to the limited immune response and complicated tumor heterogeneity [369]. It is difficult for immuno-activated cells to access the intratumor microenvironment and persist due to the immunosuppressive microenvironment and multistage physiological barriers [370]. The evolution of tumors through genetic variation allows them to evade immune surveillance and tolerate treatment [368]. Choosing appropriate tumor antigens can be challenging; however, neoantigens derived from somatic mutations present a promising approach for treating tumors with high immunogenicity. A potential method of improving cancer vaccine clinical effectiveness is the combination of immunomodulating agents with cancer vaccines in order to change the microenvironment of the tumor so that it becomes immunostimulating rather than immunosuppressive [371]. Moreover, regulatory and technical limitations prevent the implementation of particulate vaccines in clinics. These include challenges related to the evaluation of carrier pharmacokinetics and biodistribution as well as the investigation of validation techniques utilized for antigen release rates, formulation stability, and antigen selection [372]. Product developers are facing a great deal of ambiguity due to the absence of rules and harmonization from these regulatory authorities, which is impeding the creation and promotion of innovative products enabled by nanotechnology. Thus, identifying and agreeing on regulatory requirements for the tested product/device is necessary for a smooth approval process [373].

Furthermore, vaccines formulated with biomaterials have the potential to induce undesirable immune responses, resulting in inflammation and immune suppression. These shortcomings, however, can be mitigated through sustained advancements in the realms of immunology, biotechnology and materials science. Thus, an ongoing research endeavor is indispensable for the development of NP-based materials that stand as efficacious and secure components of vaccine applications.

As an integral aspect of nanovaccine design, it is imperative to account for this possibility [215, 339, 366]. Additionally, the formidable challenge of identifying tumor associated antigens persists. The customization of NPs to cater to individual patient variations presents a universal obstacle in cancer treatment [366]. The imperative lies in the creation of preclinical models and personalized medicine strategies centered around the intricate milieu of human tumors and their microenvironment. These approaches are urgently needed to facilitate more comprehensive assessments of both efficacy and safety in the context of emerging cancer nanovaccines [14, 339]. Thorough evaluation of experimental effectiveness to ensure concreteness is vital, encompassing considerations of dosage ratios and the discrepancy between animal models and cancer patients [366]. To obtain potent and durable clinical antitumor benefits, the fusion of vaccination with other forms of cancer therapy, such as chemotherapy, radiotherapy, PTT, targeted monoclonal antibody therapy, and ICB, emerges as a strategic avenue [374–380]. The negative effects of combination therapy must be fully understood prior to use. Considering large tumors have a greater resistance to immunotherapy and harbor more suppressor cells, inducing potent immune responses in the tumor microenvironment may be challenging [381]. To boost antitumor immunity, exosomes are rapidly developed as next-generation nanomedicine platforms for cancer treatment. Exosomes are used in numerous clinical investigations as an early detection tool and as prospective biomarkers for a variety of probable cancer forms [382]. Moreover, the NACHT, LRR and PYD domain-containing protein 3 (NLRP3) inflammasome may be a useful target for enhancing immunogenicity of nanovaccines [54]. Furthermore, it has been discovered that targeting metabolic reprogramming is a promising treatment strategy for cancer [383]. Recent studies also demonstrate the potential of probiotic formulations in augmenting the efficacy of cancer nanovaccines [384].

Several cutting-edge nanovaccines exhibit considerable promise for cancer immunotherapy, including personalized vaccines [134], cytomembrane nanovaccines [385], peptide-based vaccines [386, 387] and nanodiscs [388, 389]. These new nanovaccine strategies might revolutionize or improve cancer immunotherapy. Li and Wang et al. developed an innovative strategy to enhance personalized immunization by employing nanovaccines loaded with neoantigens and complemented by adoptive dendritic cell transfer. This innovative strategy involves coating cancer cell membranes with neoantigen-loaded NPs, facilitating the targeted delivery of neoantigens to resident DCs and macrophages. Through this approach, a synergistic delivery of identified neoantigens and undefined antigens derived from autologous tumor lysate is achieved, orchestrating the initiation of personalized antitumor T cell immunity [134]. Cytomembrane nanovaccines stand out by mimicking both tumor cells and antigen-presenting cells, demonstrating therapeutic efficacy in cancer treatment. Zhang and Feng et al. introduced a novel approach that utilizes reprogrammed cell membranes derived from DCs and fused cells of cancer cells as a basis for tumor vaccines. Through the fusion of these immune-associated cells, a robust expression of the complete tumor antigen complex and immunological co-stimulatory molecules on the resulting cytomembrane is achieved. By emulating APCs and cancer cells, this membrane vaccine strategy offers versatility, enabling the development of distinct vaccines tailored to various tumor types and accommodating diverse functionalities from supporters [385]. The precision of peptide-based nanovaccines is notable, contributing to enhanced accuracy, improved vaccine stability, prolonged circulation time, and minimal adverse effects. Additionally, Moon and Schwendeman et al. developed a personalized vaccine nanodisc platform based on synthetic high-density lipoprotein and found that the nanodiscs induced neoantigen-specific CTL frequencies that were up to 47 times higher than those observed with soluble vaccines and surpassed even the most potent adjuvant currently in clinical trials, such as CpG in Montanide, by a remarkable 31-fold [389]. These advancements in nanovaccine technology hold the promise of revolutionizing the vaccination landscape, potentially leading to substantial improvements in public health outcomes.

The review provides a foundation for additional research and development in this rapidly evolving field. Nanovaccines can progress global immunization efforts and promote a stronger, healthier society by overcoming challenges and seizing possibilities [390]. Successful vaccine formulation studies require an understanding of the interactions between adjuvants, antigens, and antigen delivery mechanisms. The effects of adjuvants on antigen interaction processes are not entirely known since adjuvants and antigens differ in their physicochemical properties. It will be necessary to continuously research the essential characteristics of various carriers, adjuvant activity, delivery effectiveness, and the mechanism of action of small molecules in order to produce new vaccine adjuvants and enhance vaccination formulations [391]. It is essential to carry out thorough clinical trials in order to receive regulatory approval and general acceptance. It is possible to significantly enhance immune responses through targeted administration and immunomodulation. Nanotechnology can facilitate customized and combination immunization regimens, enhancing vaccine effectiveness and meeting individual patient needs. With the development of combination vaccination, immunization has become simpler and more accessible [390].

## Conclusion

Underpinned by their distinct advantages, nanovaccines have demonstrated great potential in both prevention and treatment of cancer. This review underscores the notable strides witnessed in the evolution of NP delivery platforms for the advancement of preventive and therapeutic cancer vaccines. Nanovaccines hold the potential to revolutionize cancer treatments, providing patients with highly effective treatments with minimal side effects and improved life quality. Meanwhile, NP vaccines lay the foundation for a versatile technology with the capacity to substantially elevate public health standards and foster breakthroughs within the realm of cancer management. Nevertheless, it remains evident that challenges pertaining to the transition from laboratory innovation to clinical reality, the upscaling of manufacturing, and the attainment of regulatory approvals remain formidable challenges requiring concerted efforts. As we forge ahead, overcoming these challenges and harnessing the full potential of nanovaccines could herald a new era of innovative and effective cancer therapeutics, allowing us to take a profound leap forward in the fight against this formidable disease.

## Data Availability

All materials are available from the corresponding authors.

## Abbreviations

AgNPs: Sliver NPs
AngTE: Annona glabra L
AnsTE: Annona squamosa L
anti-PD-1: Anti-programmed cell death protein 1
APCs: Antigen-presenting cells
AuNPs: Gold nanoparticles
AuNP-Es: AuNPs coated with West Nile virus
BMDC: Bone marrow-derived dendritic cells
BP: Bisphosphonate
CAR-T: Chimeric antigen receptor T-cell
cGAS-STING: Cyclic guanosine monophosphate–adenosine monophosphate synthase-stimulator of interferon genes
COMB: Combination with HBc VLPs
CpG: Cytosine-phosphate-guanine
CRA-NPs: CpG/R848/Adpgk-codeliverying nanoparticles
CRT: Calreticulin
CTLs: Cytotoxic lymphocytes
Cur/L-OHP@HAP NPs: Hydroxyapatite NPs that were co-loaded with curcumin and L-oxaliplatin
DARPin: Encapsulin-Designed Ankyrin repeat protein
DCs: Dendritic cells
Der p1: Dermatophagoides protein 1
DNA: Deoxyribonucleic acid
DTaP: Tetanus toxoids
ELISA: Enzyme-linked immunoassay
FasL: Fas ligand
FDA: Food and Drug Administration
HBsAg: Hepatitis B antigen
HER2: Human epidermal growth factor receptor 2
HMGB1: High mobility group box protein B1
hPBMCs: Human peripheral blood mononuclear cells
ICB: Immune checkpoint blockade
IFN: Interferons
IgA: Immunoglobulin A
IgG: Immunoglobulin G
IGG: Indocyanine green
IL: Interleukin
ISCOMs: Immunostimululatin complexes
LCP: Lipid-coated calcium phosphate
MDSCs: Myeloid-derived suppressor cells
MHC: Major histocompatibility complex
MiniSOG: Mini-singlet oxygen generator
mPAP: Prostatic acid phosphatase
MSN: Mesoporous silica nanoparticles
mTHPC: Meta-tetrahydroxy-phenylchlorin
MUC1: Mucin 1
NDV: Newcastle disease viruses
neoAgs: Neoantigen peptides
NK: Natural killer
NLRP3: NACHT, LRR and PYD domain-containing protein 3
NPs: Nanoparticles
OVA: Ovalbumin
PD-1: Programmed cell death protein 1
αPD-1: An anti-programmed death-1 antibody
PDA: Polydopamine
PDT: Photodynamic therapy
PLA: Poly (lactic acid)
PLGA: Poly (lactic-co-glycolic acid)
PRRs: Pattern recognition receptors
PTT: Photothermal therapy
PTX: Paclitaxel
rCD44v: Recombinant CD44 variants
RMmAGL: Multiresponsive adjuvant nanoparticles
SEM: Scanning electron microscope
TAAs: Tumor-associated antigens
TAMs: Tumor-associated macrophages
TCR: T cell receptor
TEM: Transmission electron microscopes
Th: T helper
TLRs: Toll-like receptors
TME: Tumor microenvironment
TMT: Tumor metastasis targeting
TNBC: Triple-negative breast cancer
TRAIL: Tumor necrosis factor-related apoptosis-inducing ligand
TRP2: Tyrosinase-related protein 2
TUNEL: Terminal deoxynucleotidyl transferase dUTP nick end labelling
VLPs: Virus-like particles
